# Unraveling tumor cell heterogeneity and epithelial-mesenchymal plasticity in gastric adenocarcinoma: an integrative multi-omics framework evaluating PVR/CD155 as a tumor cell-intrinsic EMT-associated target

**DOI:** 10.3389/fimmu.2026.1932169

**Published:** 2026-09-07

**Authors:** Jianwen Li, Zitong Qin, Ting Wang, Weiping Li, Jinmin Ma, Quanlin Guan

**Affiliations:** 1 Department of Oncology Surgery, The First Hospital of Lanzhou University, Lanzhou, Gansu, China; 2 The First Clinical Medical College of Lanzhou University, Lanzhou, Gansu, China; 3 Department of Urology, The Second Hospital of Lanzhou University, Lanzhou, Gansu, China; 4 Department of Urology, The First Hospital of Lanzhou University, Lanzhou, Gansu, China; 5 Medical Frontier Innovation Research Center, The First Hospital of Lanzhou University, Lanzhou, Gansu, China

**Keywords:** cancer-associated fibroblasts, epithelial mesenchymal transition (EMT), gastric cancer, PVR/CD155, single-cell RNA sequencing

## Abstract

**Background:**

The tumor microenvironment (TME) of gastric adenocarcinoma is exceptionally heterogeneous, and epithelial-mesenchymal transition (EMT) is a central mechanism promoting local invasion and metastatic spread. Even so, how EMT-programmed cells are spatially arranged within tumor tissue, how they interact with neighboring immune populations, and which molecular nodes might be exploited therapeutically remain incompletely defined.

**Methods:**

We built a large-scale, multi-layered analytical pipeline that combined single-cell transcriptomics (approximately 250,000 cells drawn from two independent patient cohorts), Visium-based spatial transcriptomics processed through Bayesian cell2location deconvolution, niche-level SpaTopic modeling, deep-learning histology analysis (ResNet50 feature extraction paired with CellProfiler-derived morphometrics), and ensemble survival modeling spanning over one hundred algorithmic combinations. Gaussian mixture modeling was used to define EMT-high cell states, the Scissor framework was applied to link fibroblast subsets to patient mortality, and a multi-tier filtering scheme was used to nominate druggable candidate genes. The top candidate, PVR/CD155, was interrogated experimentally by RT-qPCR, Western blotting, immunohistochemistry, and siRNA knockdown in AGS cells. We further evaluated PVR druggability by molecular docking, a 100-nanosecond molecular dynamics trajectory, and MM/GBSA binding-energy estimation using PP-121 as a candidate ligand.

**Results:**

Sub-clustering identified seven fibroblast subclusters: Fib_APOD, Fib_COL4A1, Fib_SLPI, Fib_COL1A1, Fib_CCL4, Fib_STMN1, and Fib_S100B. The SLPI-high subset preferentially localized to peritoneal metastases. Phenotype-guided Scissor mapping nominated a survival-associated fibroblast program enriched within COL4A1-expressing fibroblast states. PVR/CD155 was prioritized as an EMT-linked candidate gene; single-cell expression profiling showed that PVR was most frequently detected in endothelial, epithelial and fibroblast compartments, with low detection in lymphoid and plasma cells. PVR/CD155 was significantly upregulated in gastric cancer cell lines and tumor tissues. PVR/CD155 knockdown in AGS cells decreased proliferation, migration and invasion, supporting a tumor cell-intrinsic functional role rather than establishing PVR as a stromal immune biomarker. Molecular docking and dynamics simulations identified PP-121 as a candidate PVR-binding ligand for future biochemical validation.

**Conclusion:**

The proposed framework connects single-cell resolution with spatial and histological data, produces validated prognostic tools and nominates PVR/CD155 as a tumor cell-intrinsic EMT-associated target candidate for gastric cancer. Stromal or immune regulatory roles of PVR remain plausible but require direct compartment-specific and functional validation.

## 1 Introduction

Worldwide, gastric cancer is one of the most important tumours that account for a high number of cancer deaths. According to the latest data, there are almost 968,000 new cases of gastric cancer annually and close to 660,000 deaths, making it the fifth most diagnosed cancer and a leading cause of cancer death globally (1). Patients diagnosed with locally advanced or metastatic disease continue to experience poor outcomes despite meaningful advances in surgical technique, systemic chemotherapy or immune checkpoint blockade. A principal contributor to this therapeutic barrier is the high molecular variability between individuals and the dynamic, compartmentalized nature of the gastric cancer tumor microenvironment (2, 3). The gastric TME is an ever-shifting system of malignant cells, stromal cells and immune cells whose interactions promote tumor growth and progression, create local immune tolerance, and limit therapy (4, 5). Thus, next-generation gastric cancer diagnostics and therapeutics require an extensive understanding of this cellular ecosystem and the signaling logic regulating it.

Epithelial-mesenchymal transition, or EMT, is a process involved in tumor development. As a result of epithelial-mesenchymal transition, epithelial cells lose their apical-basal polarity and detach from their neighbouring epithelial cells, through the loss of cell-cell adhesion, due to loss of E-cadherin (6, 7). Subsequently, they adopt a mesenchymal identity which is elongated and motile. Furthermore, communicating with neighbouring cells and penetrating their underlying connective tissue, this heightens their migratory and invasive capacity (6, 7). In gastric tumors, the aberrant activation of EMT programs strongly correlates with deep gastric wall invasion, peritoneal seeding, therapy resistance and decreased overall survival (8). Significantly, EMT is not a cell-autonomous event and is regulated by paracrine stromal signals, especially those from cancer-associated fibroblasts (CAF) and tumor-associated macrophages (TAM) (9, 10). Nonetheless, the localization and “address” of EMT-primed cells, alongside their niche, were not sufficiently mapped within the gastric tumor tissue.

Single-cell RNA sequencing (scRNA-seq) has greatly enhanced the ability to resolve cellular composition at high resolution (11), but this technique necessarily forfeits the native spatial context of the tissue. Spatial transcriptomics (ST) captures gene expression while preserving positional information. These two approaches are often combined by projecting cell states back to tissue coordinates using deconvolution tools such as cell2location (12, 13). Simultaneously, machine learning techniques are increasingly being used to identify robust biomarkers for oncology risk stratification (14). Most existing tools for gastric cancer prognosis rely on either transcriptomic data alone or purely qualitative pathological assessment, indicating the need for approaches that integrate multiple data modalities and scales.

To address this gap, we generated a multi-omics, multi-scale computational pipeline to reconstruct the EMT landscape of gastric cancer from scRNA-seq, spatial transcriptomics, digital pathology, and ensemble machine learning. We merged around 250,000 single-cell transcriptomes from two independent cohorts to characterize the TME cellular architecture, notably fibroblast diversity and EMT-associated cell states. We employed Gaussian mixture modeling to define high-confidence EMT-associated cell states and confirmed their spatial distribution using cell2location deconvolution and SpaTopic-based niche discovery. We used the Scissor algorithm to identify fibroblast subpopulations associated with patient prognosis. We also linked histological patterns to EMT-related transcriptional programs by analyzing deep features from ResNet50 and morphometric parameters from CellProfiler in TCGA-STAD. We constructed an ensemble model from more than 100 machine-learning combinations to develop a prognostic tool and prioritize PVR as a candidate EMT-associated molecular node. We then validated the clinical relevance of PVR by Western blotting, RT-qPCR and immunohistochemistry, which showed marked upregulation in gastric cancer cell lines and patient tumor tissue. PVR knockdown by siRNA in AGS cells significantly reduced proliferation, migration and invasion. Finally, we assessed druggability by molecular docking and 100-nanosecond all-atom molecular dynamics simulation, nominating the multi-kinase inhibitor PP-121 as a candidate small-molecule binder of PVR. Together, this multi-layered strategy links single-cell biology to translational oncology.

## 2 Materials and methods

### 2.1 Data sources

This study relied on multimodal sequencing data from four independent public data sources. Using accessions GSE183904 and GSE163558, we obtained raw single-cell transcriptomes from the Gene Expression Omnibus (GEO). These data included primary gastric tumors, matched non-tumor gastric mucosa, peritoneal metastatic lesions, and the source-cohort group annotated as Ctrl.PM. In this study, Ctrl.PM was defined as normal-appearing peritoneal tissue from patients with peritoneal metastasis, rather than as healthy donor peritoneum. Its non-malignant status was taken from the original control/normal-appearing sample annotation; the public integrated metadata available to us did not contain slide-level pathology re-review fields or an independent healthy peritoneum cohort. The two scRNA-seq GEO series were treated as independent, non-overlapping cohorts: after QC, GSE183904 contributed 151,520 cells from 40 samples derived from 31 source patients (10 Ctrl, 28 PT, 1 Ctrl.PM and 1 PM sample), whereas GSE163558 contributed 17,682 cells from four retained samples representing three source patients in the public annotation (NT/NT1, PT1, PT2 and PT3; 1 Ctrl and 3 PT samples; the full source series contains 10 samples from six patients). No GEO sample accession, integrated sample label or source patient label was shared between GSE183904 and GSE163558 in the metadata used for this analysis. The Cancer Genome Atlas (TCGA) dataset was used to retrieve bulk RNA-seq data, associated clinical annotations, and digitized H&E whole-slide images (WSIs) for stomach adenocarcinoma (STAD). Spatial transcriptomic data generated using the 10x Genomics Visium platform were downloaded from GEO accession GSE251950. The quantitative spatial revision included all four processed Visium sections available for analysis (GC1, GC4, GC7 and GC9). Detailed patient-level clinical covariates for these Visium sections were not available in the processed public metadata supplied with the downloaded objects; therefore, spatial conclusions were interpreted as within-section molecular, niche-level and spatial-statistical associations rather than clinical subgroup comparisons. Model validation relied on the fully external GSE62254 cohort. A complete sample-level post-QC count table and a cell-type-stratified UMI-threshold bias check are provided in **Supplementary Table 1**.

### 2.2 Single-Cell RNA-seq processing

We used Seurat (version 4.4.0) to process raw single-cell expression data in R. Quality control measures were employed to filter out cells with fewer than 500 unique molecular identifiers (UMIs), a mitochondrial transcript fraction greater than 25%, or fewer than 250 detected genes; genes identified in fewer than three cells were excluded. The final post-QC atlas contained 169,202 cells across 44 samples, comprising 31,093 Ctrl cells from 11 samples, 136,376 PT cells from 31 samples, 184 Ctrl.PM cells from one sample and 1,549 PM cells from one sample; dataset-level patient/source-patient counts and sample-level cell counts are listed in **Supplementary Table 2**. DoubletFinder was used to flag putative doublets computationally (15). Harmony (16) was then used to correct batch effects across patients and datasets. Following integration, the 2,000 most variable genes were selected using FindVariableFeatures, the data were scaled, and the first 30 principal components were used for downstream graph construction, clustering and UMAP visualization. To evaluate whether Harmony integration preserved biological signal while improving batch mixing, we performed integration QC on the final integrated Seurat object using UMAPs colored by sample/batch, tissue group and major cell type; sample-wise cell-type composition; canonical marker-gene expression across annotated cell types; and k-nearest-neighbor LISI-style inverse-Simpson metrics comparing the pre-integration PCA space with the post-integration Harmony embedding. Cell-type kNN purity was also calculated as a marker-preservation metric. These QC results are provided in **Supplementary Figure 1**.

Cell-type identities were assigned using well-established marker genes: epithelial cells (KRT19, CLDN4, GKN1, PGC); T cells (CD3E, CD3G, CD3D); myeloid cells/macrophages (C1QC, CSF1R, CD68); B cells (IGKC, IGHM, MS4A1, CD79B); endothelial cells (CDH5, VWF, FABP4, PLVAP); plasma cells (CD19, BANK1, BLK); fibroblasts (LUM, FGF7, DCN, COL1A1, CXCL14); and mast cells (CPA3, CST3, KIT). Sub-clustering of myeloid cells and fibroblasts was carried out using FindAllMarkers (min.pct = 0.25, logfc.threshold = 0.25), and each subcluster was named according to its distinguishing gene signature. Fibroblast re-clustering resolved seven subclusters: Fib_APOD, Fib_COL4A1, Fib_SLPI, Fib_COL1A1, Fib_CCL4, Fib_STMN1, and Fib_S100B; their name-defining marker genes are listed in **Supplementary Table 3**.

### 2.3 Tissue-level cell distribution analysis

To determine whether particular cell populations were preferentially distributed across tissue types or disease states, we calculated the ratio of observed-to-expected (Ro/e) cell counts using the epitools R package (version 10.1). Expected counts were derived from the marginal totals of cell type and sample across the entire dataset. Ro/e values were then used to flag cell populations enriched or depleted in primary tumor (PT), non-tumor gastric control (Ctrl), peritoneal metastatic lesions (PM), and normal-appearing peritoneal tissue from patients with peritoneal metastasis (Ctrl.PM). Because Ctrl.PM represents metastasis patient-derived normal-appearing peritoneum rather than healthy peritoneum, enrichment in this group was interpreted as a descriptive compositional feature and not, by itself, as evidence of a pre-metastatic niche.

### 2.4 Pseudotime and cell-state trajectory modeling

Cell-state trajectories were inferred with scTour (version 1.0.0), a deep-learning framework implemented in Python 3.8 (17). Highly variable genes specific to each population were used as model input, and scTour applied a negative binomial loss function to derive pseudotime values and vector field estimates for each cell. Pseudotime was interpreted as an inferred transcriptomic ordering from cross-sectional scRNA-seq data, not as direct evidence of chronological differentiation or an irreversible cell fate.

### 2.5 Spatial deconvolution and niche identification

Cell-type composition within individual Visium spots was estimated using cell2location, a hierarchical Bayesian deconvolution method (13), with our annotated single-cell atlas serving as the reference. The reference model was trained across 250 epochs, using genes shared between the single-cell and spatial datasets. Spatial mapping itself was run for 30,000 training iterations, and the 5th percentile of the posterior cell-abundance distribution was taken as a conservative abundance estimate.

Across GC1, GC4, GC7 and GC9, 4,992 array positions were processed per section, of which 1,882–4,244 in-tissue spots were retained for analysis. Median genes per spot ranged from 786 to 5,012, median UMI counts ranged from 1,023 to 15,128, and median mitochondrial fractions ranged from 0.46% to 2.26% (**Supplementary Table 4**). EMT pathway activity was computed from the MSigDB Hallmark gene set HALLMARK_EPITHELIAL_MESENCHYMAL_TRANSITION (systematic name M5930; MSigDB v2026.1.Hs). The complete 200-gene target list is provided in **Supplementary Table 5**, using detected genes in the spatial expression matrix. Niche-level EMT differences were tested by Kruskal-Wallis tests within each section and by pairwise Wilcoxon rank-sum tests, with Benjamini-Hochberg correction for multiple testing. Spatial autocorrelation was quantified using Moran’s I and Geary’s C with a 200-μm neighborhood radius, and EMT-niche co-localization was evaluated using Spearman spatial cross-correlation between spot EMT score and neighboring niche composition plus permutation-based high-EMT neighborhood enrichment.

Spatial niches were defined using SpaTopic, which applies Latent Dirichlet Allocation (LDA) to decompose the cell2location-derived cell-type abundance matrix into spatially coherent tissue topics (18). We set num_topics = 10 and assigned each spot to its dominant CellTopic retained in the SpaTopic output. CellTopic labels were then mapped to biological niche names according to the dominant enriched cell-type program and marker-gene profile. To make the annotation reproducible, we report for each CellTopic/niche the number and proportion of assigned spots, the median and interquartile range of the dominant topic loading, and the top niche-enriched marker genes. Abbreviated labels were standardized as Epithelial, Fibroblast, Endothelial, Immune, Infiltration, Myeloid, B cell, Plasma and Mast niches. Spatial niche plots were generated from the original Visium spot coordinates, and each spatial niche panel includes a 0.5 mm scale bar.

### 2.6 Differential expression and functional enrichment

For cell type and subtype marker gene identification, we used Seurat's FindAllMarkers function, setting a log_2_ fold_change threshold greater than 0.5 (log_2_FC > 0.5) and retaining only genes expressed in at least 25% of cells. Using this approach, we identified differentially expressed genes among the various cell types and subtypes. To further elucidate the functional characteristics of each cell state, we performed Gene Ontology (GO) enrichment analysis based on these differentially expressed gene sets to reveal the enriched biological processes and molecular functions.

### 2.7 Gaussian mixture modeling to define EMT-active cells

We implemented a model-based classification scheme to isolate high-confidence EMT-active cells with limited false positives rather than applying an arbitrary median cutoff. EMT scores were calculated from genes in the EPITHELIAL_MESENCHYMAL_TRANSITION that were detected in the single-cell expression matrix. Using the Seurat metadata and normalized expression matrix, we generated an expression matrix X, where rows represented individual cells and columns represented retained genes from this EMT gene set. The mclust R package was used to fit a two-component Gaussian mixture model to the per-cell EMT score, with the model written in plain text as:


f(s_i)=pi_1*N(s_i|mu_1,sigma_1^2)+pi_2*N(s_i|mu_2,sigma_2^2, with pi_1+pi_2=1

Here, s_i denotes the EMT score of cell i; pi_k is the mixing proportion; and mu_k and sigma_k^2 denote the mean and variance of mixture component k, respectively. Using a maximum of 100 expectation-maximization iterations, model fitting was terminated when the log-likelihood change dropped below 1e-5. The high-EMT state was assigned to the component with the greater mean EMT score. Cells were assigned high-confidence EMT-positive status if they belonged to this high-activity component. The Wilcoxon rank-sum test was then used to test differential expression between EMT-positive and EMT-negative cells, and the top resulting genes were taken forward for prognostic modeling.

High-confidence EMT-positive and EMT-negative cells were balanced by down-sampling, 80% of each class was assigned to the training set, and the remaining cells were split evenly into test set 1 and test set 2. The top 100 differentially expressed genes between EMT-high and EMT-low cells were used as predictors. The 12 evaluated algorithms were logistic regression, linear discriminant analysis, AdaBoost, decision tree, random forest, XGBoost, ridge regression, lasso regression, elastic-net regression, support vector machine, stepwise logistic regression, and naive Bayes. Complete package/function calls, tuning grids, probability cutoffs, cross-validation settings and model-selection rules are provided in **Supplementary Table 6**.

Uncertainty for the SVM calibration and decision-curve analyses was estimated by nonparametric bootstrap resampling of the test-set prediction/output pairs with 1,000 resamples. For calibration plots, 95% confidence intervals were calculated for the observed positive fraction within each predicted-risk bin.

### 2.8 Linking single-cell subpopulations to clinical phenotypes

The Scissor algorithm was used as a phenotype-guided mapping approach to relate fibroblast single-cell transcriptomes to TCGA-STAD overall survival, rather than as an independent functional screen. The bulk input was Matrix.scissor.csv, containing 373 TCGA-STAD samples with matched expression and clinical information. Overall survival phenotype was defined from Clinic.cssv using futime as continuous follow-up time and fustat as vital status (1 = death event, 0 = censored; 137 events and 236 censored cases; median follow-up time, 416 days). Because Scissor was run in Cox survival mode (family = “cox”), no dichotomized binary survival phenotype, deceased/alive time point, or censoring cutoff was introduced; the phenotype entered the model as Surv(futime, fustat). The Scissor phenotype, input dimensions, gene-matching rule and final parameters are summarized in **Supplementary Table 7**. The fibroblast single-cell input contained 16,753 cells. Bulk and single-cell matrices were matched by gene symbol, and only genes present in both matrices were retained, yielding 17,786 shared genes for model fitting. Genes absent from either dataset were excluded from the Scissor input. Scissor first quantile-normalized the combined bulk and single-cell expression matrix, then calculated Pearson correlations between each TCGA bulk sample and each fibroblast single cell to construct the phenotype-to-cell similarity matrix. The model was fitted using the single-cell shared-nearest-neighbor graph and graph-regularized Cox regression. The alpha parameter was fixed *a priori* at 0.05 for the final analysis rather than optimized *post hoc* across outcome results, and the fitted model returned lambda = 0.0242013. Cells with positive Cox coefficients were labeled Scissor+ and interpreted as associated with poor prognosis/higher mortality risk; cells with negative Cox coefficients were labeled Scissor-, and all other cells were treated as background. Therefore, Scissor+ cells were interpreted as a survival-associated fibroblast transcriptional state under TCGA-STAD phenotype guidance, not as proof that the cells themselves causally determine poor prognosis. Because Scissor returns selected cell coefficients rather than independent cell-level P values, coefficient sign and downstream enrichment were used for interpretation; ssGSEA scoring of the Scissor+ signature in TCGA-STAD was used as a cohort-level consistency analysis.

To evaluate robustness of the Scissor output, we examined the selected-cell coefficient direction, fibroblast subtype enrichment, patient/sample-level distribution of Scissor+ cells and cohort-level survival consistency of the Scissor+ signature. Specifically, we summarized the percentage of Scissor+ cells within each fibroblast subtype for every available patient/sample and visualized these values as a patient-level heatmap. Scissor coefficients were interpreted by sign and selected-cell status rather than by independent cell-level p values, because the graph-regularized Cox model returns coupled selected cell coefficients and does not provide valid independent per-cell p values or FDR-adjusted q values for each selected single cell. We also recognized that bulk-level survival associations of a fibroblast-derived signature may be confounded by overall stromal or immune content. Therefore, additional validation should include multivariable Cox models adjusted for clinical stage and ESTIMATE-derived stromal and immune scores in TCGA-STAD, followed by validation of the Scissor+ signature in an independent external gastric cancer cohort such as GSE62254.

### 2.9 Cell-cell communication analysis

Ligand-receptor communication patterns were reconstructed using NicheNet (version 2.2.0) (19), which draws on curated prior-knowledge networks encompassing ligand-receptor pairs, ligand-target relationships, and intracellular signaling weights. For this analysis, the NicheNet target gene set was explicitly defined as the EPITHELIAL_MESENCHYMAL_TRANSITION entry from the Human MSigDB Hallmark gene set HALLMARK_EPITHELIAL_MESENCHYMAL_TRANSITION (systematic name M5930; MSigDB v2026.1.Hs).

### 2.10 Digital pathology feature extraction

Histological features were extracted from TCGA-STAD H&E whole-slide images to connect tissue-level architecture with molecular phenotype. In total, 373 patients with complete survival information were included. Each slide was divided into non-overlapping tiles, with tiles containing artifacts (ink marks, folds, mounting defects) excluded. Remaining tiles were passed through a pre-trained ResNet50 convolutional neural network to generate a 2,048-dimensional deep feature vector per tile.

In a parallel workflow, interpretable morphometric measurements were obtained using CellProfiler (20). Color tiles were first converted to grayscale via ColorToGray and then separated into hematoxylin and eosin channels using UnmixColors. Global-level descriptors of texture, granularity, and co-localization were computed, and at the single-object level, nuclear and cellular boundaries were segmented to extract measurements of size, shape, staining intensity, and texture. Per-tile summary statistics (mean, median, standard deviation) were compiled to give a comprehensive morphometric profile for each patch.

### 2.11 Ensemble machine learning for prognostic modeling

Candidate genes from the MSigDB Hallmark EMT pathway were first screened for significant correlation with the deep-learning and morphometric image features. The TCGA-STAD cohort (n = 373) was divided into an 80% training subset and a 20% internal test subset, with GSE62254 reserved entirely for external validation.

Prognostic models were built using the Mime ensemble framework in R, which integrates ten core survival algorithms (21) and combines them into more than 100 distinct model configurations. The configuration achieving the highest mean concordance index (C-index) across all cohorts was selected as the final model.

Kaplan-Meier curves generated with the survminer package were used to compare survival between high- and low-risk groups, with statistical significance assessed by the log-rank test (P < 0.05). Time-dependent receiver operating characteristic (ROC) curves quantified 1-, 3-, and 5-year prediction accuracy and were benchmarked against conventional clinical staging. A prognostic nomogram was subsequently constructed using the rms package.

### 2.12 PVR spatial immunoregulatory co-localization analysis

To evaluate whether the available spatial transcriptomic data supported a stromal-immune interpretation of PVR/CD155, we analyzed all four Visium sections (GC1, GC4, GC7 and GC9) for PVR, its immune receptors TIGIT, CD226 and CD96, and T-cell/exhaustion markers. Target-gene presence was confirmed in the SCT assay for PVR, TIGIT, CD226, CD96, PDCD1, CTLA4, HAVCR2, LAG3, TOX, ENTPD1, CXCL13, CD3D, CD3E, CD8A, CD8B, GZMB and PRF1 in every section. We calculated spot-level Spearman correlations between PVR and individual receptors or module scores, including a PVR-receptor score and an exhausted T-cell score. We also evaluated 200-um neighborhood correlations between spot PVR expression and neighboring receptor/exhaustion scores, using the Visium center-to-center distance approximation from the processed spatial coordinates. Spatial autocorrelation of PVR, receptor-score and exhausted T-cell score was assessed using Moran’s I. These analyses were interpreted as spatial association tests only and were not treated as functional evidence that stromal PVR modulates immune-cell activity.

To avoid conflating epithelial EMT with immune or stromal activation programs, we interpret this score in a compartment-aware manner. In epithelial/tumor cells, high HALLMARK_EPITHELIAL_MESENCHYMAL_TRANSITION activity is interpreted as EMT or epithelial-mesenchymal plasticity. In fibroblast, endothelial, myeloid or immune-enriched compartments, the same score is described as EMT-associated or mesenchymal-like gene-set activity, because shared genes such as VIM, FN1 and S100A-family members can also mark stromal remodeling or immune activation; immune cells were not interpreted as undergoing epithelial-mesenchymal transition.

### 2.13 Cell lines and siRNA transfection

The non-tumorigenic gastric epithelial line GES-1 and five gastric cancer lines (HGC27, AGS, MKN45, MKN1, SNU216) were cultured under standard conditions. AGS cells, which showed the highest endogenous PVR expression, were selected for functional knockdown experiments and were transfected with three independent PVR-targeting siRNAs (siRNA1, siRNA2, siRNA3) or a non-targeting control (NC) using Lipofectamine RNAiMAX. Knockdown efficiency was assessed by Western blot at 48 hours post-transfection, and the two most efficient constructs (siRNA2 and siRNA3) were carried forward for functional assays. Sequence details appear in **Supplementary Table 8**.

### 2.14 Cell proliferation assay

Proliferation was measured with the CCK-8 colorimetric assay. NC-, siRNA2-, and siRNA3-transfected AGS cells were plated in 96-well plates at 2 × 10³ cells per well. At 0, 24, 48, and 72 hours, 10 μL of CCK-8 reagent was added per well followed by a 2-hour incubation at 37 °C, and absorbance was read at 450 nm. Assays were run in technical triplicate across three independent biological repeats.

### 2.15 Wound healing (scratch) assay

AGS cells transfected as above were grown to roughly 90% confluence in six-well plates. A straight scratch was made using a sterile 200 μL pipette tip, detached cells were rinsed away with PBS, and the medium was switched to serum-free RPMI-1640. Wound closure was imaged by phase-contrast microscopy at 0, 24, and 48 hours.

### 2.16 Transwell migration and invasion assays

Directional migration and invasion capacity were assessed using Corning Transwell inserts. For migration assays, 2 × 10^4^ cells suspended in 200 μL serum-free medium were seeded into the upper chamber, while 600 μL of complete medium containing 20% FBS served as a chemoattractant in the lower chamber. After 24 hours at 37 °C, cells remaining on the upper membrane surface were removed with cotton swabs. Cells that had migrated to the lower surface were fixed with 4% paraformaldehyde (15 minutes), stained with 0.1% crystal violet (20 minutes), and counted under light microscopy.

### 2.17 Western blot analysis

Total protein was extracted using ice-cold RIPA buffer containing protease/phosphatase inhibitors, and protein concentration was determined via BCA assay. Equal amounts of protein (20–30 μg/lane) were resolved on 10% SDS-PAGE gels and transferred onto PVDF membranes. Membranes were blocked for 1 hour with 5% non-fat milk in TBST, then incubated overnight at 4 °C with anti-PVR/CD155 primary antibody (Proteintech, 27486-1-AP), using ACTB or GAPDH as loading controls. After washing, HRP-conjugated secondary antibodies were applied for 1 hour at room temperature, and bands were visualized by enhanced chemiluminescence.

### 2.18 Quantitative real-time PCR

Total RNA was isolated with TRIzol reagent, and its concentration and purity were verified by NanoDrop spectrophotometry. One microgram of RNA was reverse-transcribed to cDNA using the PrimeScript RT Reagent Kit. Real-time PCR was performed in triplicate using SYBR Green Master Mix on a Bio-Rad CFX96 instrument. Relative expression was calculated with the 2^(-ΔΔCt) method, and primer sequences are listed in **Supplementary Table 8**.

### 2.19 Immunohistochemistry

Paired tumor and adjacent non-tumor mucosal tissue from three gastric cancer patients were collected with informed consent and institutional ethical approval. Four-micrometer sections were deparaffinized, rehydrated, and subjected to heat-induced antigen retrieval in citrate buffer (pH 6.0, 10 minutes). Endogenous peroxidase activity was quenched with 3% hydrogen peroxide (10 minutes), and non-specific binding was blocked with 5% normal goat serum (30 minutes). Sections were incubated overnight at 4 °C with anti-PVR/CD155 antibody (Proteintech, 27486-1-AP), followed by HRP-conjugated secondary antibody (1 hour, room temperature). Signal was developed using DAB substrate, and slides were counterstained with hematoxylin, dehydrated, and mounted for evaluation.

### 2.20 Molecular docking

The three-dimensional structure of human PVR/CD155 was obtained from the AlphaFold Protein Structure Database, and the PP-121 ligand structure was retrieved from PubChem and geometry-optimized with the MMFF94 force field. Docking calculations were performed in AutoDock Vina (version 1.2.3) (22). Waters, ions, and any co-crystallized ligands were stripped from the receptor using PyMOL (version 2.5.2), and both receptor and ligand were converted to PDBQT format with ADFRsuite (version 1.0) (23). A grid box encompassing the extracellular face of PVR was defined, and docking was carried out with an exhaustiveness value of 32; the highest-scoring pose was retained for structural interpretation.

### 2.21 Molecular dynamics simulation

All-atom molecular dynamics of the PVR-PP-121 complex were carried out in AMBER 24. AM1-BCC charges were assigned to PP-121 via the Antechamber module, and the ligand and protein were described using the GAFF2 and ff14SB force fields, respectively (24). The complex was solvated in a cubic TIP3P water box, with K+ and Cl– ions added for charge neutrality. Energy minimization proceeded through 2,500 steps of steepest descent followed by 2,500 steps of conjugate gradient. The system was gradually heated from 0 K to 298.15 K over 200 ps under NVT conditions, followed by 500 ps of NVT equilibration and 500 ps of NPT equilibration at 298.15 K and 1 atm. Production dynamics were then run for 100 ns under NPT conditions with periodic boundary conditions. Non-bonded interactions used a 10 Å cutoff, long-range electrostatics were handled via Particle Mesh Ewald (PME), hydrogen bonds were constrained with SHAKE, temperature was controlled via a Langevin thermostat (collision frequency 2 ps^-^¹), and trajectory frames were saved every 10 ps (25).

### 2.22 MM/GBSA binding free energy calculation

The binding free energy of the PVR-PP-121 complex was calculated using MM/GBSA for the 90–100 ns range of the trajectory. The total binding free energy (ΔG_bind) was partitioned as follows:


$$\Delta G_{bind}=\Delta {\text{G}}_{\text{complex}}-(\Delta {\text{G}}_{\text{receptor}}+\Delta {\text{G}}_{\text{ligand}})$$

$$=\Delta {\text{E}}_{\text{internal}}+\Delta {\text{E}}_{\text{VDW}}+\Delta {\text{E}}_{\text{elec}}+\Delta {\text{G}}_{\text{GB}}+\Delta {\text{G}}_{\text{SA}}$$

where ΔE_internal captures bond, angle, and dihedral terms; ΔE_VDW represents van der Waals contributions; ΔE_elec represents electrostatic contributions; ΔG_GB is the polar solvation term computed with a generalized Born model (26); and ΔG_SA is the non-polar solvation term derived from solvent-accessible surface area (SASA). Entropic terms were not evaluated because of their computational burden and limited certainty. We also performed per-residue decomposition to identify key residues contributing most to binding.

### 2.23 Statistical analysis

All statistical analysis was performed in R (version 4.2.0). Wilcoxon rank-sum test was used for group-wise transcriptional comparisons. Kaplan-Meier method with log-rank comparisons was used to analyze survival. Pearson or Spearman coefficients were used to assess correlations. All tests were two-sided and a P < 0.05 was taken as statistically significant.

## 3 Results

### 3.1 A Single-cell atlas of the gastric tumor microenvironment

**Figure 1:**
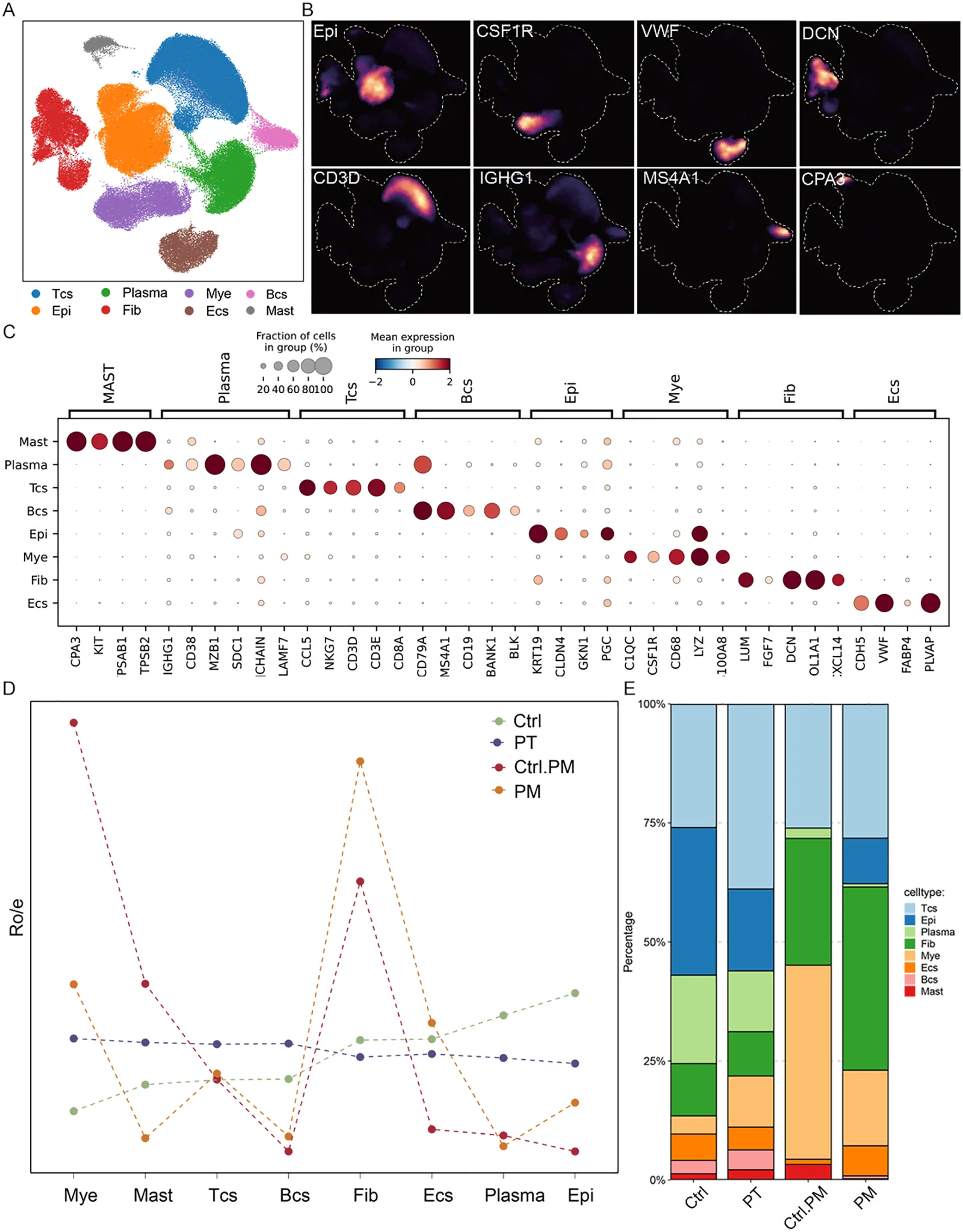
Integrated single-cell atlas reveals the cellular landscape of gastric cancer. **(A)** UMAP plot showing eight major cell types. Each dot represents a single cell, colored by cluster (Tcs, Plasma, Mye, Bcs, Epi, Fib, Ecs, and Mast). **(B)** UMAP feature plots displaying the expression distribution of characteristic marker genes corresponding to the cell types shown in **(A)**. **(C)** Dot plot illustrating the expression profiles (mean expression level and percentage of positive cells) of representative marker genes in each identified cell type in gastric cancer. **(D)** Line plot depicting the distribution bias of Ro/e scores across different sample groups: Ctrl (non-tumor gastric control), PT (primary tumor), Ctrl.PM (normal-appearing peritoneal tissue from patients with peritoneal metastasis; not healthy donor peritoneum), and PM (peritoneal metastatic lesion). **(E)** Stacked bar chart showing the percentage of each cell type in the indicated sample groups (Ctrl, PT, Ctrl.PM, PM).

We integrated roughly 250,000 single-cell transcriptomes from two independent cohorts (GSE183904 and GSE163558) to characterize the cellular landscape of gastric tumors. After quality filtering and Harmony-based batch correction, the final integrated object used for integration QC contained 169,202 cells, one RNA assay, and PCA, Harmony and UMAP reductions. Graph-based clustering resolved eight major cell lineages (**Figure 1A**). Integration QC showed improved sample-level mixing after Harmony while preserving major biological structure: the sample iLISI-style median increased from 4.55 in the PCA space (IQR, 2.11-7.26) to 8.49 in the Harmony embedding (IQR, 5.84-10.71), whereas median cell-type kNN purity remained 1.00 before and after Harmony integration. UMAPs colored by sample/batch, tissue group and cell type, together with sample-wise composition plots and canonical marker dot plots, further supported batch mixing without loss of major cell-type marker identity (**Supplementary Figure 1**). Cluster identities were assigned by cross-referencing marker gene expression with canonical cell-type signatures, and the distribution of these markers across the UMAP embedding confirmed cluster identity (**Figures 1A, B**). The eight populations comprised epithelial cells (Epi), T cells (Tcs), B cells (Bcs), myeloid cells (Mye), fibroblasts (Fib), endothelial cells (Ecs), plasma cells, and mast cells. A dot plot summarizing marker expression and detection rates across clusters further supported these annotations (**Figure 1C**).

Ro/e-based analysis of tissue distribution showed that myeloid cells and fibroblasts were enriched within Ctrl.PM, defined here as normal-appearing peritoneal tissue from patients with peritoneal metastasis (**Figure 1D**). Because no independent healthy peritoneal scRNA-seq control was available, this enrichment may reflect the baseline stromal and myeloid composition of peritoneal tissue in patients with metastasis and should not be interpreted as direct evidence of a pre-metastatic niche. Cell-fraction analysis further described the cellular composition of the peritoneal metastatic microenvironment, with myeloid cells representing a dominant population in PM samples (**Figure 1E**). Given the recognized contribution of stromal fibroblasts to tumor invasion and extracellular matrix remodeling, we next examined fibroblast heterogeneity in greater depth.

### 3.2 Fibroblast subtypes and pseudotime-associated state structure

**Figure 2:**
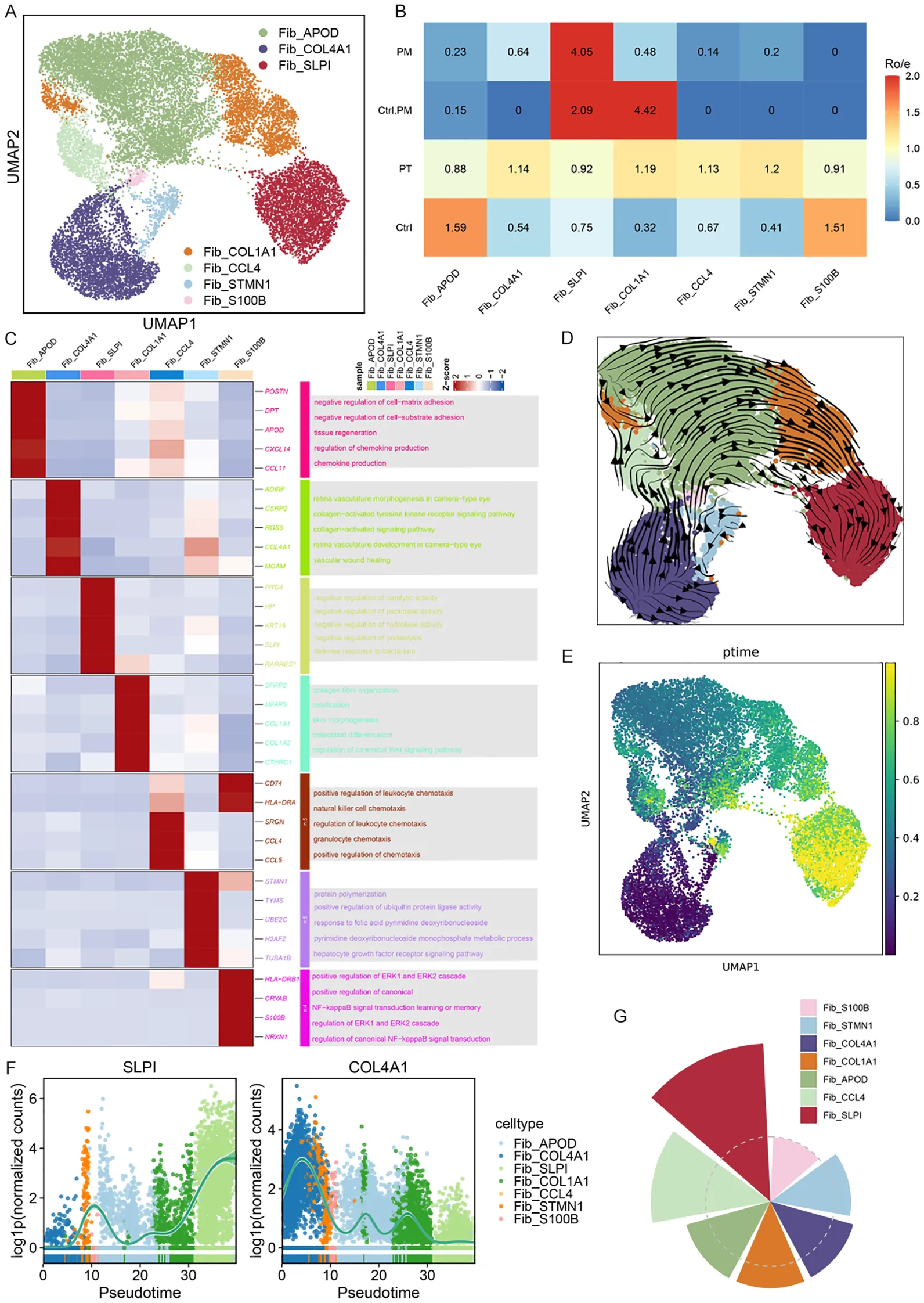
Fibroblast heterogeneity reveals seven functionally distinct subpopulations in gastric cancer. **(A)** UMAP plot of fibroblast subclusters: Fib_APOD, Fib_COL4A1, Fib_SLPI, Fib_COL1A1, Fib_CCL4, Fib_STMN1, and Fib_S100B. **(B)** Heatmap showing Ro/e scores of subclusters across different sample groups: Ctrl (non-tumor gastric control), PT (primary tumor), Ctrl.PM (normal-appearing peritoneal tissue from patients with peritoneal metastasis; not healthy donor peritoneum), and PM (peritoneal metastatic lesion). **(C)** Heatmap of marker gene expression with corresponding GO enrichment analysis on the right. **(D)** Inferred fibroblast cell-state trajectory vector field. **(E)** UMAP plot colored by inferred pseudotime (ptime). **(F)** Slingshot pseudotime expression dynamics of SLPI and COL4A1 across fibroblast subclusters. **(G)** Pie/rose diagram illustrating the proportion/specificity of each fibroblast subcluster.

Computational isolation of fibroblasts and high-resolution re-clustering segregated seven subsets, annotated as Fib_APOD, Fib_COL4A1, Fib_SLPI, Fib_COL1A1, Fib_CCL4, Fib_STMN1, and Fib_S100B according to their top marker genes from FindAllMarkers (**Figure 2A**; **Supplementary Table 3**). Ro/e analysis across the harmonized clinical groups indicated strong enrichment of the Fib_SLPI subpopulation in PM lesions, supporting an association with peritoneal metastatic tissue (**Figure 2B**). Ctrl.PM was retained as a separate normal-appearing peritoneal comparator from metastasis patients, but its fibroblast composition was not used as standalone evidence for a pre-metastatic niche. Heatmap-based visualization of marker genes and GO enrichment showed distinct functional signatures of each subcluster (**Figure 2C**).

Using scTour for trajectory reconstruction, the inferred vector field (**Figure 2D**) and pseudotime-colored UMAP (**Figure 2E**) indicated that Fib_SLPI cells occupied a late-pseudotime region of the inferred fibroblast state continuum. Rather than establishing a fixed differentiation endpoint, this pattern supports an activated SLPI-high fibroblast state, or late-pseudotime-associated state, linked to peritoneal metastatic tissue. An independent Slingshot analysis showed broadly consistent pseudotime-associated changes in SLPI and COL4A1 expression, but these inferred trajectories cannot determine real-time lineage directionality from cross-sectional scRNA-seq data (**Figure 2F**). Among the seven fibroblast subsets, Fib_SLPI, Fib_COL4A1, and Fib_CCL4 showed high subtype specificity across datasets (**Figure 2G**).

After describing the diversity of fibroblasts at the single-cell level, we next analysed how these states are organized in intact tissue.

Across-section statistical analysis supported niche-dependent EMT activity rather than a representative-image-only pattern. Kruskal-Wallis tests for EMT score across niches gave: GC1: H = 1556.68, q=<1e-300; GC4: H = 2009.44, q=<1e-300; GC7: H = 421.16, q=7.45e-90; GC9: H = 2068.66, q=<1e-300. EMT spatial autocorrelation was quantified by Moran’s I (GC1: I = 0.616, q=0.010; GC4: I = 0.550, q=0.010; GC7: I = 0.568, q=0.010; GC9: I = 0.657, q=0.010) and Geary’s C (GC1: C = 0.391, q=0.010; GC4: C = 0.450, q=0.010; GC7: C = 0.421, q=0.010; GC9: C = 0.335, q=0.010). Spatial cross-correlation and neighborhood enrichment further evaluated EMT co-localization with niche structure; the strongest EMT-neighbor niche associations were GC9 Epithelial rho=-0.549, q=1.96e-273; GC4 Fibroblast rho=0.534, q=1.68e-310; GC1 Immune rho=0.510, q=9.11e-252; GC1 Epithelial rho=-0.442, q=3.54e-182, and high-EMT neighborhood enrichments were GC9 Epithelial log2 enrichment=-4.303, q=0.011; GC7 Mast log2 enrichment=-2.768, q=0.011; GC1 Myeloid log2 enrichment=1.987, q=0.011; GC1 Immune log2 enrichment=1.676, q=0.011. In total, 40 cell abundance/topic-loading niche comparisons remained significant at BH-adjusted q < 0.05 (**Supplementary Figure 2**).

### 3.3 Spatial mapping of the tumor architecture using bayesian deconvolution

**Figure 3:**
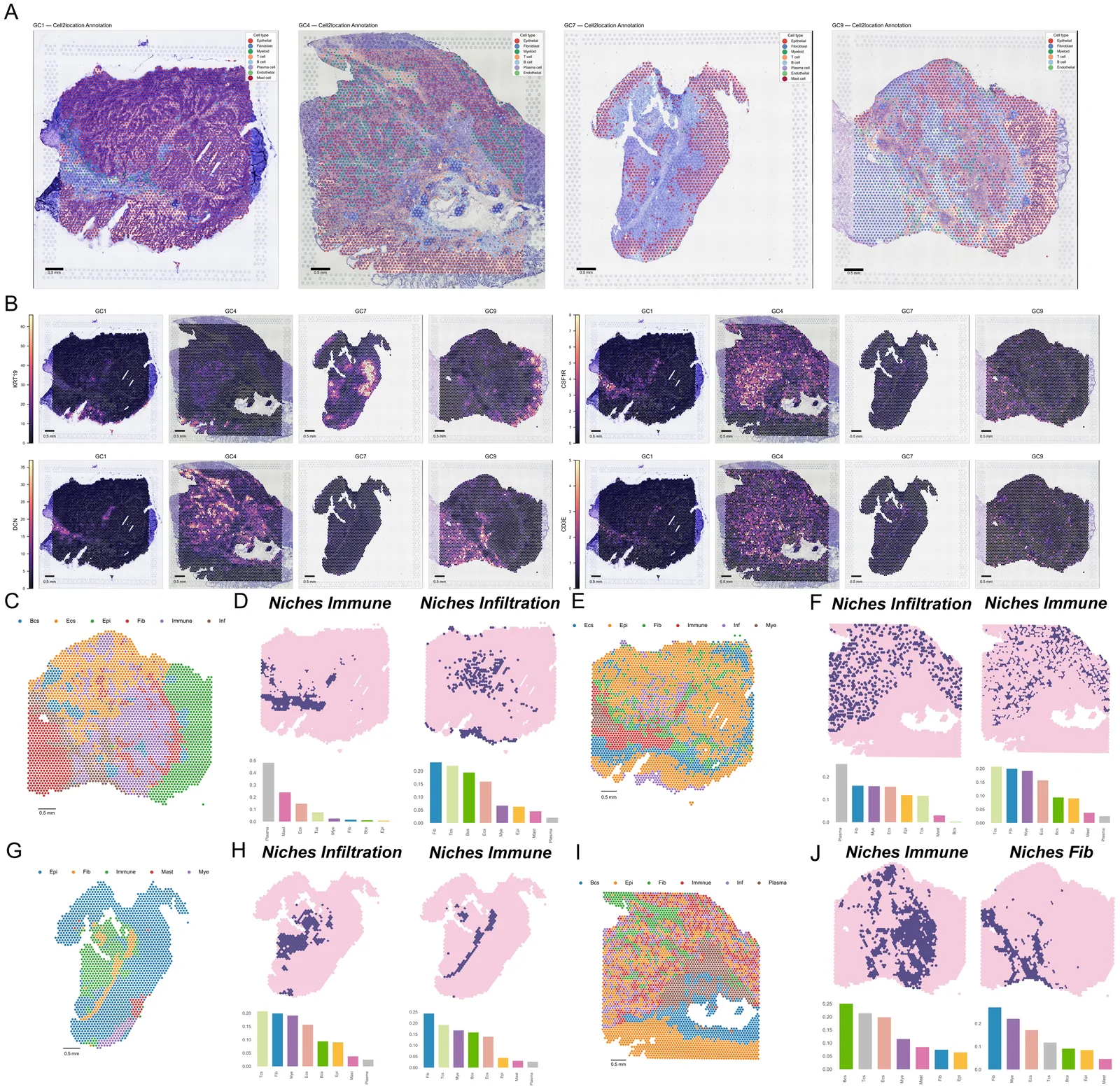
Identification of cell types and spatial niches by Cell2location deconvolution and SpaTopic modeling. **(A)** Cell type deconvolution results based on the Cell2location algorithm, with spatial spots colored according to estimated cell type abundances. **(B)** Spatial distribution and expression patterns of specific cell markers utilized to characterize distinct tissue regions. **(C, E, G, I)** SpaTopic spatial niche maps for GC1, GC4, GC7 and GC9, respectively. Niche labels were assigned using quantitative CellTopic-to-niche mapping, including assigned spot proportions, dominant topic-loading median/IQR and top marker genes. Scale bar, 0.5 mm. **(D, F, H, J)** Bar charts indicating the relative proportion of various cell types comprising each identified niche within the corresponding samples.

We employed the validated deconvolution tool Cell2location anchored to our annotated single-cell reference to map single-cell lineages on spatial transcriptomic data. The reference model was run for 250 epochs to obtain expression signatures for 15 cell types, and spatial mapping was run for 30,000 epochs to estimate per-spot cell abundances (**Figure 3A**). A visualization of four representative lineages and direct mapping of lineage-specific marker genes onto tissue sections agreed strongly with cell2location abundance estimates (**Figure 3B**).

We employed SpaTopic, which uses Latent Dirichlet Allocation to decompose the cell2location abundance matrix into spatially coherent cellular neighborhoods. To avoid subjective niche naming, we re-examined the SpaTopic output from all four Visium sections and annotated each niche using dominant CellTopic assignment, spot proportion, dominant topic-loading distribution and niche-enriched marker genes. The original abbreviated labels were standardized as Epithelial, Fibroblast, Endothelial, Immune, Infiltration, Myeloid, B cell, Plasma and Mast niches. In this framework, the Immune niche was defined by immune-enriched CellTopic assignments and accounted for 8.2%, 16.5%, 19.9% and 24.4% of spots in GC1, GC4, GC7 and GC9, respectively. The Infiltration niche was detected in GC1, GC4 and GC9, accounting for 8.3%, 14.5% and 10.0% of spots, respectively. Quantitatively, the Immune label corresponded to immune-enriched CellTopics with assigned spot fractions of 8.24% in GC1 (CellTopic5, median dominant loading 0.673), 16.54% in GC4 (CellTopic2, 0.610), 19.87% in GC7 (CellTopic2 plus CellTopic7, 0.658 and 0.661), and 24.41% in GC9 (CellTopic1, 0.603). The Infiltration label corresponded to CellTopic7 in GC1 (8.35%, median loading 0.655), CellTopic3 in GC4 (14.51%, 0.648), and CellTopic4 in GC9 (10.00%, 0.712). Niche-enriched genes supporting these labels are listed in the revised supplementary tables; the Immune niche included CXCL5 and immunoglobulin-related genes (IGHGP, IGLC1, IGLC3, IGHG1, and IGLC2), whereas the Infiltration niche included CFD, SFRP2, CCDC80, SERPINF1, PI16, EFEMP1, TNXB, EPCAM, FBN1, and LUM. A comprehensive overview of CellTopic composition and niche assignment across all four sections is provided in **Supplementary Figure 3**.

Because epithelial-mesenchymal transition (EMT) is known to drive both tumor invasion and immune remodeling, we asked how EMT pathway activity relates to these spatial niches.

### 3.4 Machine learning identification of EMT gene-set-high cell states

**Figure 4:**
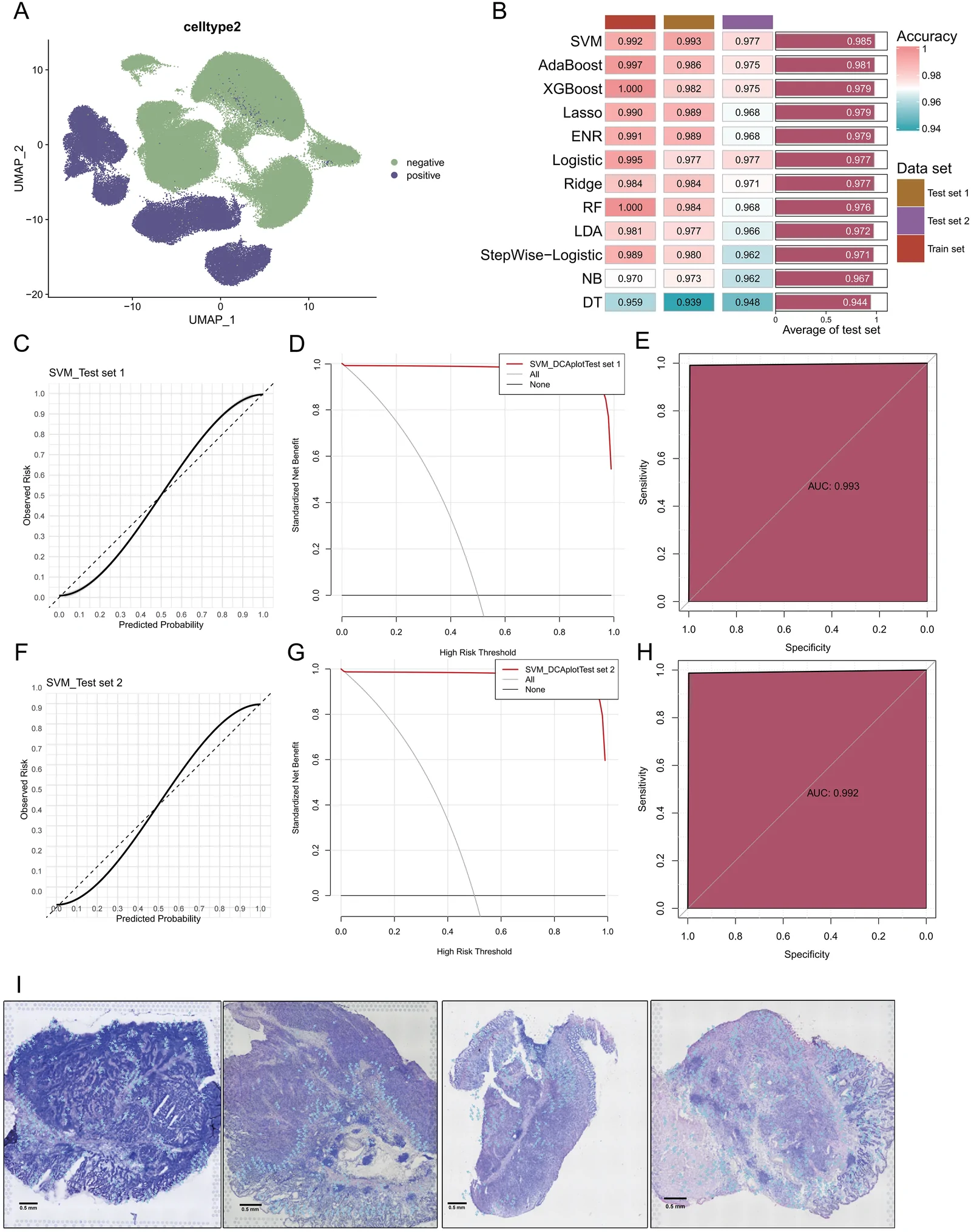
Identification of EMT pathway-associated subpopulation cells. **(A)** UMAP plot showing the distribution of positive (blue) and negative (green) cells identified by machine learning algorithms. **(B)** Heatmap displaying the predictive accuracy of 12 machine learning models across the train set, test set 1, and test set 2. **(C, D)** Calibration curves evaluating the alignment between predicted probabilities and observed risks for the SVM model in test set 1 **(C)** and test set 2 **(D)**. **(E, F)** Decision curve analysis showing the net benefit of the prediction model (red line) compared to "treat all" (black line) and "treat none" (gray line) strategies across different risk thresholds for test set 1 **(E)** and test set 2 **(F)**. **(G, H)** ROC curves demonstrating the predictive accuracy, with AUC values of 0.993 and 0.992 for test set 1 **(G)** and test set 2 **(H)**, respectively. **(I)** Representative histological staining images of corresponding tissue sections.

To avoid the pitfalls of an arbitrary median-based threshold, we implemented a model-based classification pipeline for MSigDB Hallmark EMT gene-set activity (**Figure 4A**). A Gaussian mixture model was first used to nominate high-confidence EMT gene-set-high cells, and this classification was then cross-validated using 12 distinct machine learning algorithms across a training partition and two independent test partitions of the single-cell dataset (**Figure 4B**). Elevated EMT gene-set activity was observed in fibroblast, myeloid and endothelial compartments; outside epithelial/tumor cells, this was interpreted as EMT-associated or mesenchymal-like transcriptional activity rather than bona fide epithelial-mesenchymal transition.

Among the 12 algorithms tested, the support vector machine (SVM) classifier achieved the best overall performance. Calibration plots and decision curve analysis (DCA) confirmed robust, stable performance for the optimized SVM model across all validation partitions (**Figures 4C–F**), and ROC analysis produced very high discriminative accuracy, with AUC values of 0.993 and 0.992 in the two validation subsets (**Figures 4G, H**). SpaTopic-based niche quantification showed that EMT gene-set activity was enriched within quantitatively defined Immune and Infiltration niches, with niche labels supported by dominant CellTopic loading, spot proportions and niche-enriched marker genes (**Figures 3C–J**). This spatial signal is interpreted as niche-level enrichment of an EMT-associated/mesenchymal-like gene-expression program, not as evidence that immune cells undergo EMT. Representative H&E-stained tissue sections corresponding to the spatial transcriptomic samples are shown in **Figure 4I**.

Given that elevated EMT gene-set activity was observed within the heterogeneous fibroblast compartment, we next asked which fibroblast subpopulations carried prognostic significance.

### 3.5 Identifying fibroblast subpopulations linked to patient outcome

**Figure 5:**
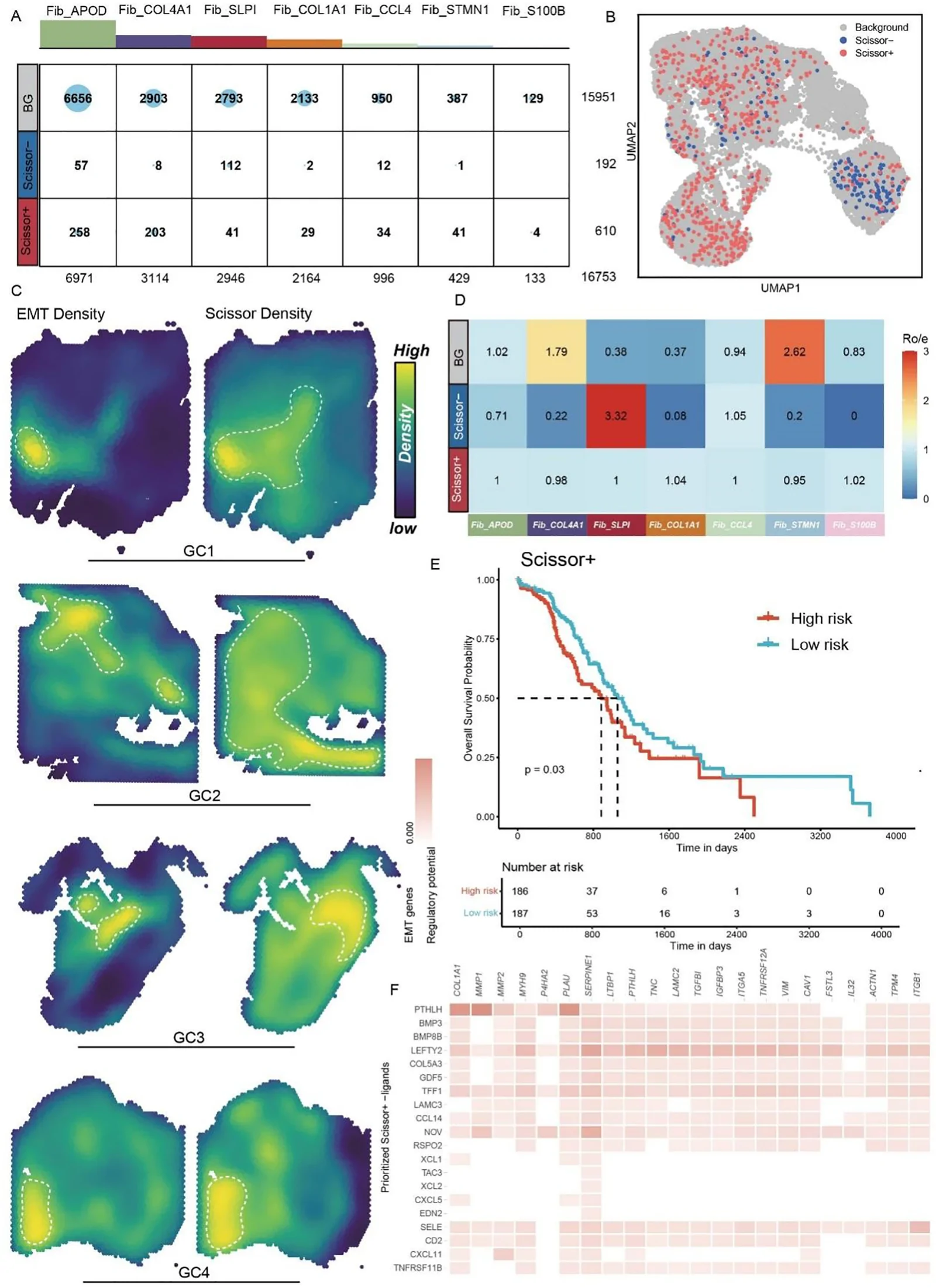
Phenotype-guided mapping of survival-associated fibroblast programs using the Scissor algorithm. **(A)** Dot plot table showing fibroblast subpopulations identified by Scissor. Scissor+ indicates cells with positive Cox coefficients in the TCGA-STAD survival-guided mapping and is associated with poor prognosis/higher mortality risk; Scissor- indicates cells with negative coefficients, and BG represents background cells not selected by the model. These labels indicate survival association rather than direct causal evidence. **(B)** UMAP plot visualizing the distribution of Scissor+, Scissor-, and Background cells. **(C)** Spatial density plots showing spatial co-localization between the MSigDB Hallmark EMT gene signature (left) and Scissor+ cell density (right) across different spatial groups (GC1-GC4). This co-localization should be interpreted as spatial association, not proof of causal regulation of EMT by Scissor+ or Fib_COL4A1 fibroblasts. **(D)** Heatmap displaying the distribution preference (Ro/e) of each fibroblast subtype among Scissor+, Scissor-, and BG groups. **(E)** Kaplan-Meier survival curves for patients stratified by the Scissor+ fibroblast signature score (p = 0.03). **(F)** NicheNet cell-cell communication analysis showing candidate regulatory links between prioritized Scissor+ ligands and EMT-related target genes; these links are hypothesis-generating and require perturbation validation. The revised Scissor figure set also includes a patient/sample-level heatmap showing Scissor+ cell percentages within each fibroblast subtype across individual cases.

We applied Scissor to map fibroblast single-cell states to TCGA-STAD survival phenotype in a Cox-model framework. Among 16,753 fibroblasts, Scissor identified 610 Scissor+ cells (positive Cox coefficients; associated with poor prognosis and higher mortality risk) and 192 Scissor- cells (negative Cox coefficients), with the remaining cells assigned to background (**Figures 5A, B**). Scissor+ cells were distributed across several fibroblast subclusters, including Fib_APOD (258 cells, 3.70% of the subcluster), Fib_COL4A1 (203 cells, 6.52%), Fib_STMN1 (41 cells, 9.56%) and Fib_CCL4 (34 cells, 3.41%). Thus, the analysis prioritized a survival-associated fibroblast program that was prominent in the COL4A1-expressing compartment but was not restricted to a single fibroblast subset. Spatially, the density of Scissor+ fibroblasts co-localized with regions of elevated EMT pathway activity, supporting spatial proximity between the survival-linked fibroblast program and EMT-active tissue states (**Figures 5C, D**). This spatial association should not be interpreted as evidence of a causal effect of Fib_COL4A1 or Scissor+ fibroblasts on EMT.

We additionally generated a patient/sample-level Scissor+ fibroblast heatmap to visualize inter-case heterogeneity. This heatmap reports the percentage of Scissor+ cells within each fibroblast subtype for each sample and shows that the survival-associated Scissor+ program is heterogeneously distributed across individual cases rather than driven by a single aggregated fibroblast pool. Consistent with the Scissor output structure, robustness was assessed using coefficient direction, subtype enrichment, patient-level distribution and ssGSEA-based cohort-level survival consistency rather than by reporting independent per-cell p values.

We next summarized the Scissor+ fibroblast program at the patient level using ssGSEA in the TCGA-STAD bulk cohort. Patients with higher Scissor+ signature scores had shorter overall survival than those with lower scores (**Figure 5E**), supporting internal consistency between the Scissor-derived cell program and the survival phenotype used for mapping. Because the Scissor model itself was trained with TCGA-STAD survival information, this analysis should be viewed as phenotype-linked validation within the same clinical resource rather than as external validation. NicheNet-based communication modeling between Scissor+ fibroblasts and EMT-active cells further nominated candidate ligand-receptor interactions that may be associated with stromal programs and EMT activity; these interactions remain hypothesis-generating and require experimental perturbation, co-culture validation or spatially resolved functional assays before causal regulation can be claimed (**Figure 5F**).

We have therefore reframed the Scissor result as phenotype-guided prioritization rather than an independent functional discovery. Because Scissor was explicitly trained on TCGA-STAD survival phenotype, the Scissor+ fibroblast program should be interpreted as a survival-associated mapping result that requires external functional validation, not as proof that Fib_COL4A1 fibroblasts causally drive poor prognosis or directly regulate EMT-active tumor cells.

We then asked whether these molecular signals could be inferred non-invasively from standard histopathology images, a step needed to translate these findings into a clinically practical tool.

### 3.6 Deep learning and morphometric analysis of histopathology

**Figure 6:**
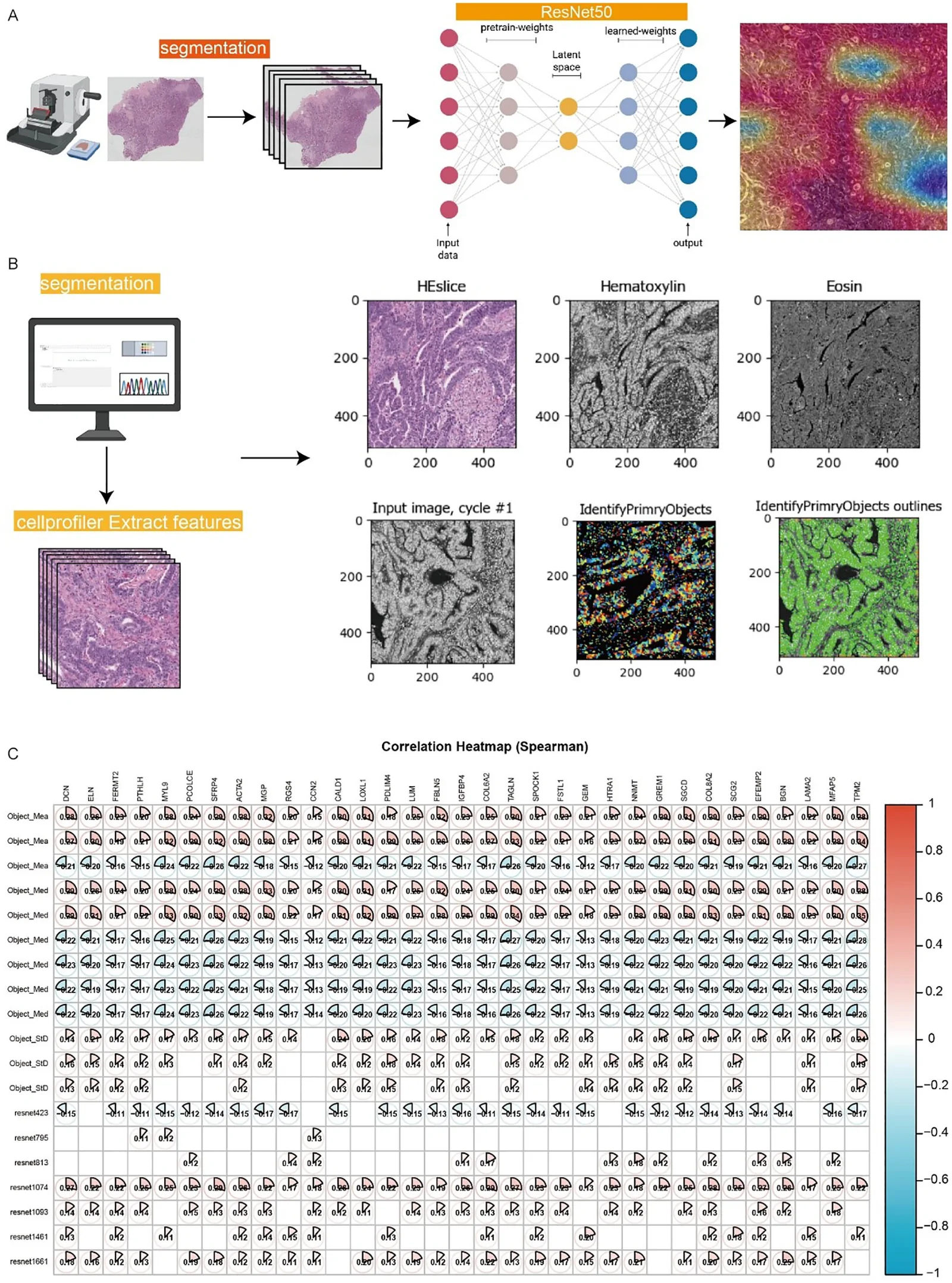
Pathological feature extraction and correlation with EMT pathway activity. **(A)** Workflow of ResNet50 for deep learning feature extraction. Whole slide images are first segmented into patches. These patches are then input into the model for feature extraction within a latent space, utilizing pretrained weights. The final output generates tables and heatmaps highlighting the spatial regions of interest for the extracted features. **(B)** Workflow of CellProfiler for pathological feature extraction. The segmented patches from **(A)** are processed, starting with the conversion of patches into grayscale images representing the hematoxylin and eosin channels. Subsequently, quantitative features, including texture, cell localization, and morphology, are calculated and output as tabular data. **(C)** Spearman correlation heatmap displaying the relationship between MSigDB Hallmark EMT gene-set expression and the pathological features (morphological and deep learning features) extracted from workflows **(A, B)**. The color scale indicates the correlation strength, revealing associations between gene expression levels and tissue morphological characteristics.

We examined H&E whole-slide images from 373 TCGA-STAD patients with complete outcome data to relate tissue morphology to underlying molecular states. The slides were partitioned into non-overlapping tiles, and artifact-containing or damaged regions were excluded automatically. A pre-trained ResNet50 network calculated deep feature vectors for each tile, and these vectors were then mapped back to their spatial origin (**Figure 6A**). This process resulted in 2,048 deep-learning-derived features for each tile (**Supplementary Table 9**). We used CellProfiler to generate interpretable morphometric descriptors from the same tiles. Images were converted to grayscale, and hematoxylin/eosin channels were separated for downstream analysis. Shape, size, nuclear-boundary and texture measurements were quantified at global and single-object levels (**Figure 6B**, **Supplementary Table 10**). Correlation analysis between image-derived features and genes from the MSigDB Hallmark EMT set identified the 33 genes with the strongest associations, summarized in a correlation matrix (**Figure 6C**).

### 3.7 Building and validating a histology-based prognostic model

**Figure 7:**
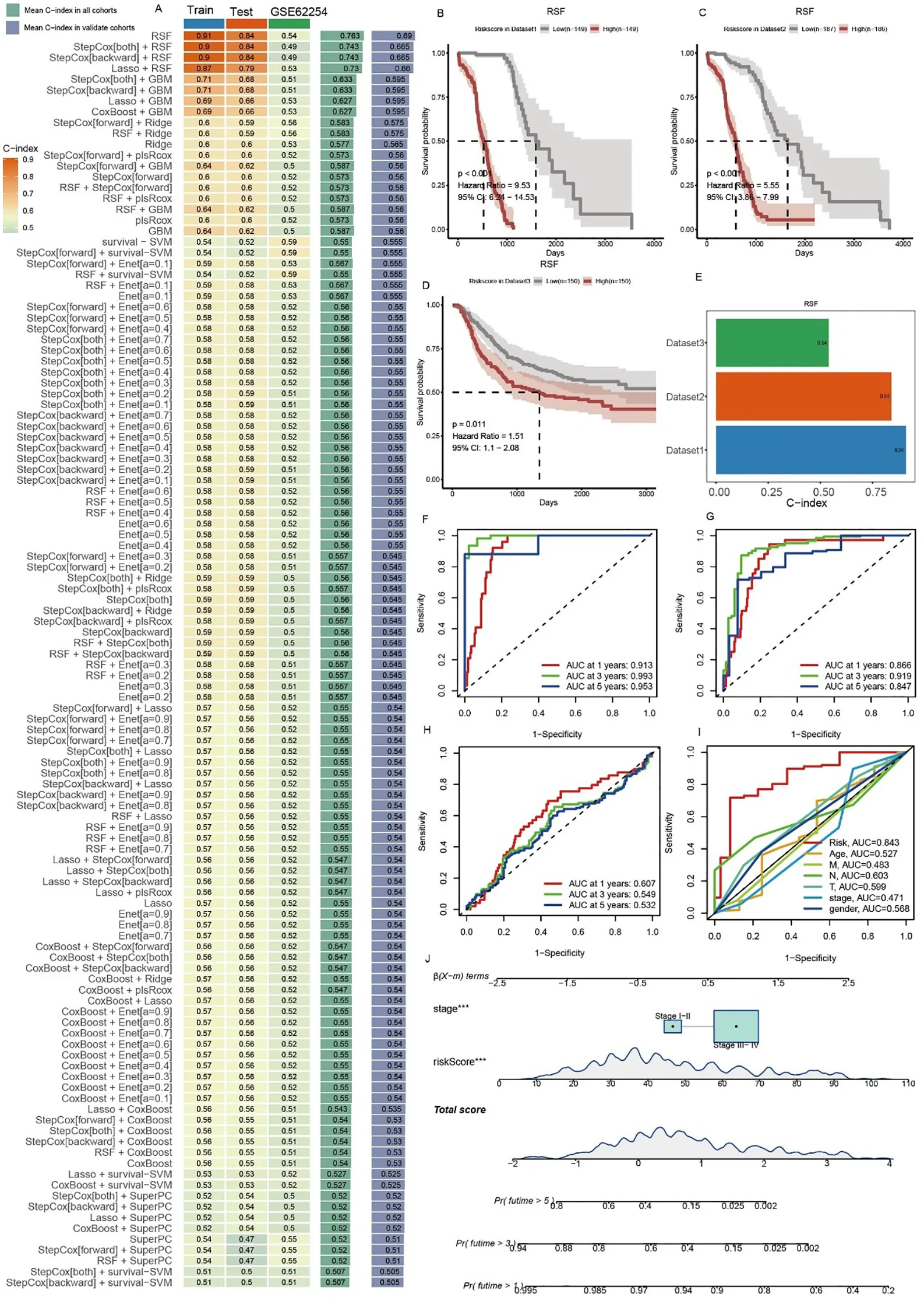
Establishment of a prognostic model based on pathological features. **(A)** Ranked bar plot displaying the C-index of various machine learning models across different cohorts, ordered by their average C-index. **(B-D)** Kaplan-Meier survival analysis for high- and low-risk patients stratified by the RSF model in the training cohort (B, p<0.001), the TCGA validation cohort (C, p<0.001), and the GSE62254 validation cohort (D, p=0.011). **(E)** Bar chart illustrating the C-index of the RSF model across the three cohorts. **(F-H)** Time-dependent ROC curves for predicting 1-, 3-, and 5-year survival in the training cohort **(F)**, the TCGA validation cohort **(G)**, and the GSE62254 validation cohort **(H)**. **(I)** ROC curves comparing the predictive performance of the risk score against other clinical pathological characteristics. **(J)** Nomogram incorporating clinical stage and risk score for predicting the probability of 1-, 3-, and 5-year OS.

Genes that correlated significantly with histological image features were carried forward as candidate model inputs. The TCGA-STAD dataset (n = 373) was partitioned into an 80% training set (n = 298) and a 20% internal test set (n = 75), and GSE62254 was retained as an external validation cohort. We used the Mime framework to evaluate more than 100 machine-learning combinations built from 10 base survival algorithms, aiming to maximize predictive accuracy and reduce overfitting. Among these models, the random survival forest (RSF) model showed the strongest and most stable C-index values across cohorts (**Figures 7A, E**).

Kaplan-Meier stratification showed that the RSF model effectively separated patients into risk groups. Overall survival was significantly better in the low-risk group than in the high-risk group (log-rank P < 0.05) (**Figure 7B**), and the same pattern was observed in both validation datasets (**Figures 7C, D**). Time-dependent ROC analysis confirmed accurate prediction at 1, 3 and 5 years (**Figures 7F–H**). Moreover, multivariable analysis showed that the RSF-derived risk score retained prognostic value beyond conventional clinical stage (**Figure 7I**). A composite nomogram integrating risk score and staging information was developed for individualized patient prediction (**Figure 7J**).

To identify candidate EMT-associated genes, we compared three distinct gene sets: differentially expressed genes derived from the GMM-defined EMT-high cells, marker genes of the survival-associated Scissor+ fibroblast program, and MSigDB Hallmark EMT genes linked with digital pathology features. PVR (poliovirus receptor, CD155) emerged at this intersection and was taken forward as a candidate EMT-associated gene for tumor-cell functional validation, rather than as *a priori* evidence for a stromal immune biomarker or a spatially inferred causal regulator.

Accordingly, PVR/CD155 was not interpreted as a direct downstream effector of the Fib_COL4A1 or Scissor+ fibroblast state. Its nomination came from an independent multi-filter prioritization strategy that intersected EMT-high cell differentially expressed genes, Scissor+ program-associated markers and pathology-linked MSigDB Hallmark EMT genes, followed by direct assessment of PVR expression and loss-of-function experiments in gastric cancer cells. We therefore present PVR as a tumor cell-intrinsic EMT-associated candidate target rather than as a mechanistically validated product of Fib_COL4A1-to-tumor paracrine signaling. Spatial co-localization analysis across all four Visium sections revealed that PVR expression positively correlated with TIGIT/CD226/CD96 immune receptor scores and with T-cell exhaustion markers at both spot and 200-μm neighborhood levels, supporting a potential stromal-immune spatial context for PVR (**Supplementary Figure 4**).

### 3.8 Experimental validation of PVR/CD155 function in gastric cancer

**Figure 8:**
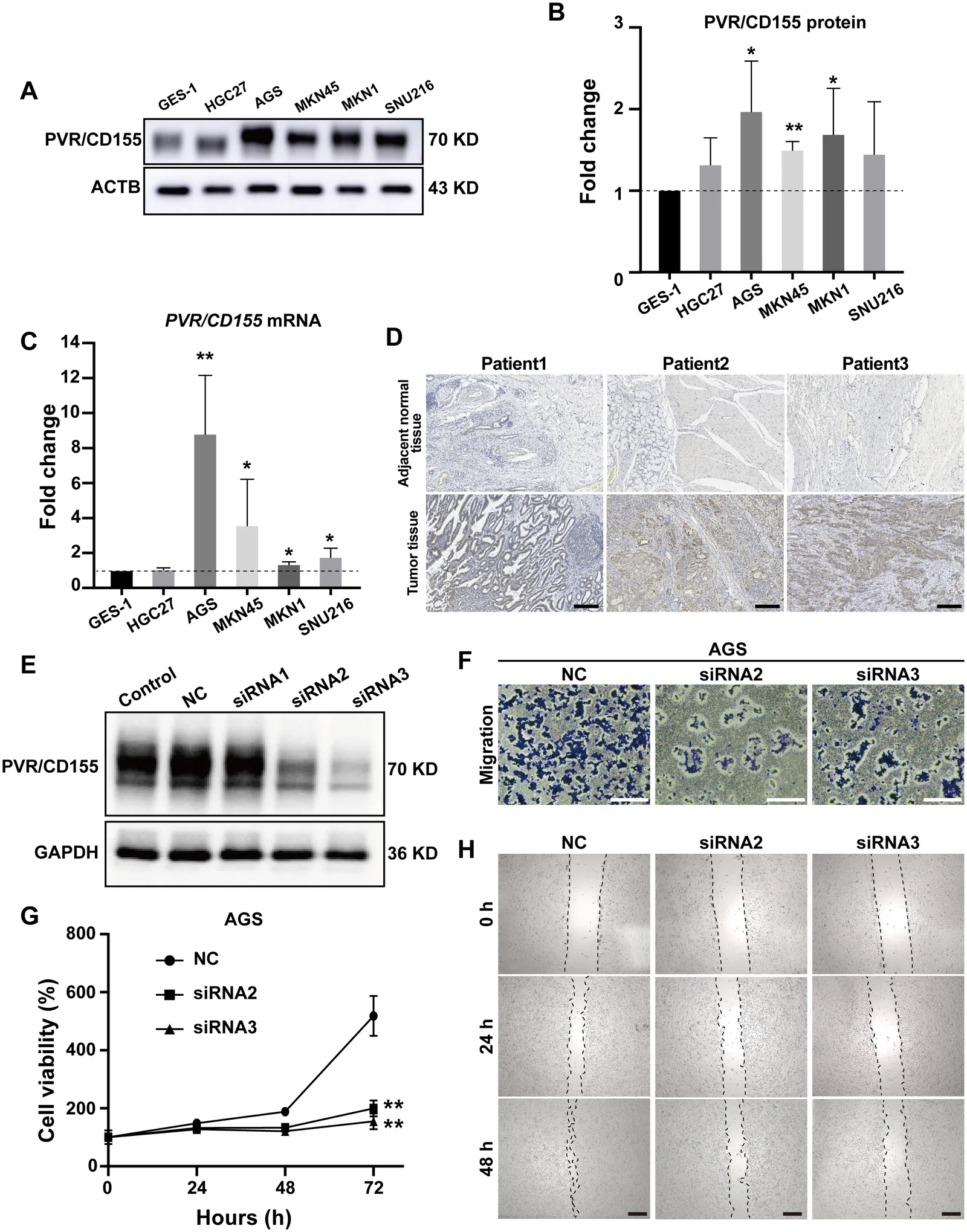
High expression of PVR/CD155 in gastric cancer cells and the suppressive effects of its knockdown on cell viability and migration. **(A)** Western blotting analysis of PVR/CD155 protein expression levels in normal gastric epithelial cells (GES-1) and indicated gastric cancer cell lines (HGC27, AGS, MKN45, MKN1, SNU216). ACTB was used as an internal loading control. **(B)** Quantification of the relative PVR/CD155 protein levels shown in **(A)**. Data represent the mean ± SD from three independent Western blotting experiments (*P < 0.05, **P < 0.01 compared to GES-1). **(C)** RT-qPCR analysis of PVR/CD155 mRNA expression levels in the indicated cell lines. The fold change was normalized to GES-1 using ACTB as an internal control. Data are presented as mean ± SD from three independent replicates (*P < 0.05, **P < 0.01). **(D)** Representative IHC staining of PVR/CD155 in adjacent normal gastric tissues and tumor tissues from three gastric cancer patients (Patient 1-3,scale bars: [200] μm). **(E)** Validation of PVR/CD155 knockdown efficiency in AGS cells. Cells were transfected with NC siRNA or three target siRNAs (siRNA1, siRNA2, siRNA3). Protein expression was analyzed by Western blotting, with GAPDH used as the loading control. **(F)** Transwell migration assays showing the migration ability of AGS cells following transfection with NC, siRNA2, or siRNA3. Cells that migrated to the lower surface were stained blue(scale bars: [100] μm). **(G)** CCK-8 assay assessing cell viability of AGS cells at 0, 24, 48, and 72 hours post-transfection with NC, siRNA2, or siRNA3. **P < 0.01 compared to the NC group. **(H)** Representative images of the scratch wound healing assay evaluating the migration capacity of AGS cells at 0, 24, and 48 hours following the indicated siRNA treatments(scale bars: [100] μm). Dotted lines indicate the boundaries of the wound.

We first evaluated the cellular context of PVR expression in the single-cell atlas. PVR was detected most frequently in endothelial cells (13.8% PVR+), epithelial cells (12.9%) and fibroblasts (9.9%), whereas detection was low in myeloid cells (3.5%), mast cells (0.8%), T cells (0.3%), B cells (0.2%) and plasma cells (0.3%). This pattern did not support describing PVR as a broad stromal immune biomarker. To further test whether PVR showed spatial proximity to immunoregulatory receptor or exhausted T-cell signals, we analyzed all four Visium sections for PVR, TIGIT, CD226, CD96 and T-cell exhaustion markers. Spot-level correlations were weak overall: the median Spearman rho was 0.02 for the PVR-receptor score (range, -0.01 to 0.06), 0.04 for the exhausted T-cell score (range, -0.01 to 0.06), 0.05 for CD96 (range, -0.01 to 0.09), approximately 0.00 for CD226 (range, -0.03 to 0.04), and approximately 0.00 for TIGIT (range, -0.03 to 0.03). The 200-um neighborhood analyses were similarly weak, with median rho values of 0.04 for neighboring receptor score (range, 0.00 to 0.11) and 0.04 for neighboring exhausted T-cell score (range, -0.04 to 0.16). PVR itself showed limited spatial autocorrelation (median Moran’s I, 0.02; range, 0.01-0.11), whereas exhausted T-cell score showed somewhat stronger spatial structure (median Moran’s I, 0.18; range, 0.05-0.30). These results support at most limited and section-dependent spatial association, not a functional stromal-immune biomarker claim. We therefore focused the experimental validation on tumor-cell intrinsic PVR function. Western blotting indicated that PVR protein levels were elevated in all five malignant gastric lines (HGC27, AGS, MKN45, MKN1, SNU216) relative to the non-tumorigenic gastric epithelial line GES-1 (**Figures 8A, B**). This was corroborated by RT-qPCR quantification of PVR transcript levels (**Figure 8C**). Immunohistochemical analysis of three paired tumor/adjacent-normal tissues confirmed higher tumor-associated PVR staining, with strong diffuse cytoplasmic and membranous staining in tumor tissue and minimal background signal in adjacent normal mucosa (**Figure 8D**).

We performed siRNA knockdown in AGS cells, which showed the highest basal PVR expression among the gastric cancer cell lines tested. Three independent siRNAs (siRNA1-3) and a non-targeting control (NC) were evaluated. Effective silencing by siRNA2 and siRNA3 was confirmed by Western blotting and RT-qPCR (**Figure 8E**, **Supplementary Figure 6A**), and these two siRNAs were used in subsequent functional assays.

CCK-8 assays showed that AGS cell proliferation was significantly inhibited after PVR knockdown from 24 to 72 hours (**Figure 8G**). In scratch assays, PVR-silenced cells retained significantly larger wound gaps at 24 and 48 hours than control cells, indicating impaired migratory ability after knockdown (**Figure 8H**). Transwell assays independently confirmed that the number of migrating cells decreased significantly after PVR silencing (**Figure 8F**). Quantitative analyses of the wound-healing and Transwell assays further confirmed that PVR knockdown significantly reduced cell migration and wound-closure rates (**Supplementary Figure 7**). Transwell assays independently confirmed that the number of migrating cells decreased significantly after PVR silencing (**Figure 8F**). Mechanistically, PVR/CD155 knockdown significantly upregulated the epithelial marker E-cadherin and downregulated the mesenchymal markers N-cadherin, vimentin, and SNAIL, indicating that PVR silencing partially reverses the EMT phenotype in AGS cells (**Supplementary Figures 6B–E**). Collectively, these findings indicate that PVR promotes gastric cancer cell growth and migration.

### 3.9 In silico docking and dynamics analysis of PVR-PP-121 interaction

**Figure 9:**
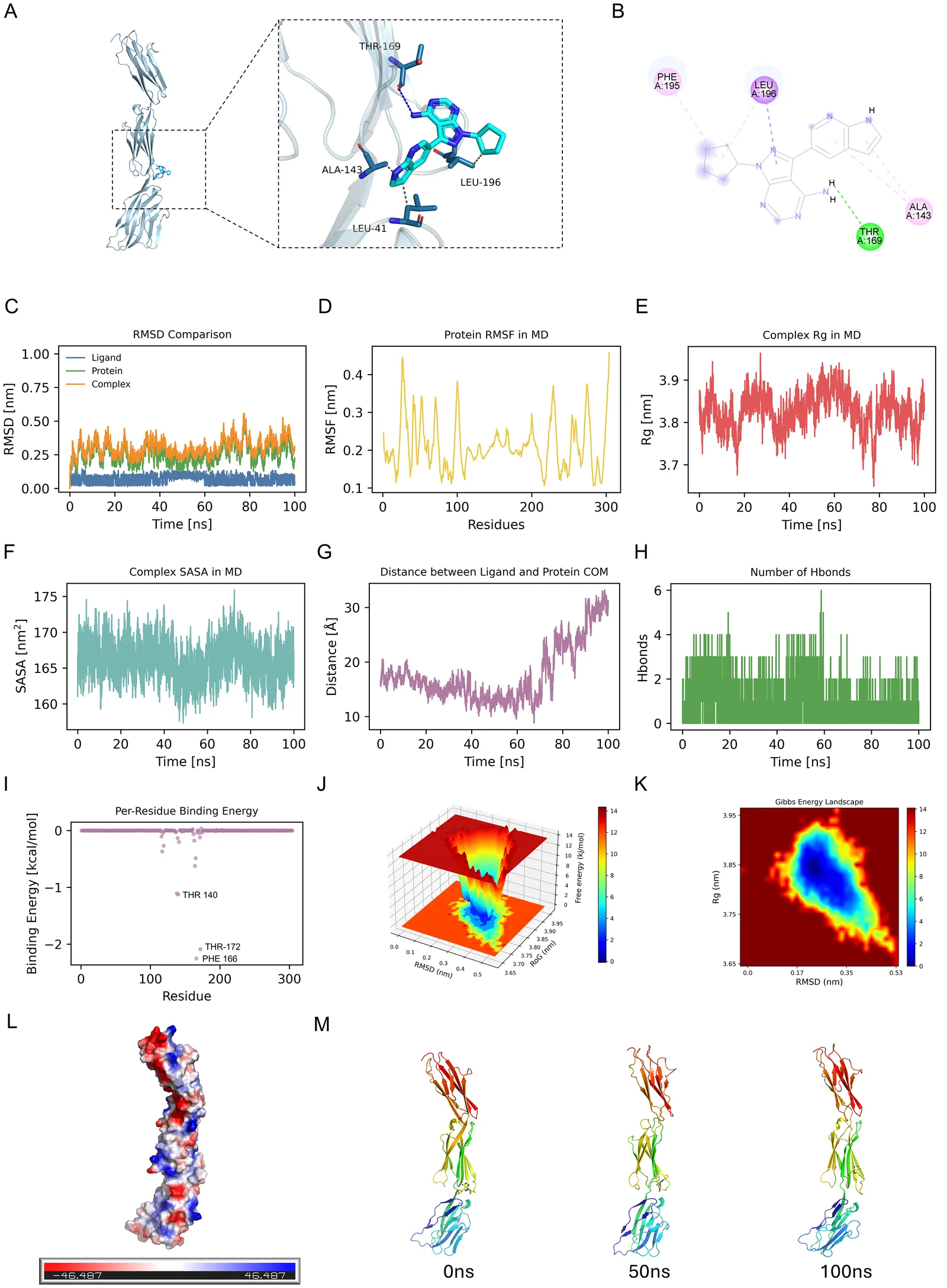
Molecular docking and molecular dynamics simulation analysis of the PVR–PP-121 complex. **(A)** Overall binding conformation of PP-121 within the PVR pocket; the magnified view on the right shows the key residue–ligand interactions. PVR is shown as a blue cartoon and PP-121 as yellow sticks; green dashed lines indicate hydrogen bonds and grey dashed lines indicate hydrophobic contacts. **(B)** Two-dimensional schematic of the ligand–residue interaction network. **(C)** RMSD profiles of the ligand, receptor, and complex over the 100 ns MD trajectory. **(D)** Per-residue RMSF distribution of the protein backbone. **(E)** Radius of gyration (Rg) of the complex as a function of time. **(F)** Solvent accessible surface area (SASA) of the complex as a function of time. **(G)** Center-of-mass distance between the ligand and the protein during the trajectory. **(H)** Number of proteins–ligand hydrogen bonds along the trajectory. **(I)** Per-residue decomposition of the binding free energy. **(J)** Three-dimensional FEL constructed from RMSD and Rg. **(K)** Two-dimensional projection of the FEL. **(L)** Electrostatic potential distribution on the PVR surface around the PP-121 binding site. **(M)** Overlay of complex conformations at 0 ns, 50 ns, and 100 ns of the MD simulation.

To evaluate the druggability of PVR, we screened PP-121 and seven structurally related compounds against the AlphaFold-predicted PVR structure using molecular docking (**Supplementary Figure 5**). Predicted binding affinities ranged from -5.493 to -8.219 kcal/mol (**Table 1**). Although PP-121 was not the top-scoring compound (-6.585 kcal/mol), it was selected for molecular dynamics simulation because it had a clear pharmacological profile, reported anti-tumor activity and suitable geometry within the hydrophobic pocket of PVR. In the best docking pose, PP-121 occupied a hydrophobic pocket on PVR, forming stabilizing hydrophobic contacts with ALA-143, LEU-196 and LEU-41, as well as a hydrogen bond with THR-169 (**Figures 9A, B**).

**Table 1:** Molecular docking scores of candidate compounds with AF-PVR protein (kcal/mol).

Target	Ligand	Docking_score(kcal/mol)
AF-PVR	(2E)-N-(4-((3-bromophenyl)amino)quinazolin-6-yl)-4-(dimethylamino)but-2-enamide	-5.893
AF-PVR	1-(1-(3-aminophenyl)-3-tert-butyl-1H-pyrazol-5-yl)-3-phenylurea	-6.222
AF-PVR	1-(1-methylethyl)-3-quinolin-6-yl-1H-pyrazolo[3-4-d]pyrimidin-4-amine	-6.421
AF-PVR	1-cyclobutyl-3-(3-4-dimethoxyphenyl)-1H-pyrazolo[3-4-d]pyrimidin-4-amine	-6.012
AF-PVR	4-((4-Methyl-1-piperazinyl)methyl)-N-(3-((4-(3-pyridinyl)-2-pyrimidinyl)amino)phenyl)-benzamide	-8.219
AF-PVR	KX-01	-7.637
AF-PVR	N-(4-Phenylamino-quinazolin-6-YL)-acrylamide	-5.493
AF-PVR	PP-121	**-6.585**

We used a 100 ns all-atom molecular dynamics simulation to investigate the stability of this complex under near-physiological conditions. The ligand RMSD remained within approximately 0.05-0.12 nm, and the protein backbone RMSD stabilized around 0.20-0.35 nm, indicating that the complex reached equilibrium without extensive structural rearrangement (**Figure 9C**). Overall RMSF values remained low during the simulation (~0.15-0.25 nm), with increased fluctuation limited mainly to flexible surface loops outside the ligand-binding region (**Figure 9D**). The radius of gyration (Rg, ~3.75-3.90 nm) and solvent-accessible surface area (SASA, ~160–172 nm2) showed no large variations along the trajectory, indicating overall stability (**Figures 9E, F**).

The center-of-mass distance between ligand and receptor was relatively stable initially but began to drift after approximately 70 ns, indicating minor repositioning of PP-121 in the binding pocket during the second half of the simulation (**Figure 9G**). During the trajectory, the number of hydrogen bonds varied from 1 to 4 (**Figure 9H**). Per-residue free-energy decomposition indicated that a discrete subset of interface residues contributed favorably to binding (**Figure 9I**). The free-energy landscape (FEL) constructed from RMSD and Rg coordinates showed a single, well-defined energy minimum, consistent with a thermodynamically stable bound conformation (**Figures 9J, K**). Surface charge distributions were complementary to the binding-interface charge and were consistent with specific ligand binding (**Figure 9L**). Structural snapshots at 0, 50 and 100 ns indicated that the PVR core remained largely rigid, whereas PP-121 shifted position within a nearby surface groove while preserving hydrophobic and hydrogen-bond interactions, reflecting conformational flexibility of the ligand (**Figure 9M**).

The total binding free energy (ΔG_bind) over the sTable 90–100 ns window estimated by MM/GBSA was -14.02 ± 1.74 kcal/mol (**Table 2**). Binding was driven primarily by favorable van der Waals interactions (-20.78 ± 2.37 kcal/mol) and electrostatic contributions (-15.16 ± 2.17 kcal/mol), which outweighed the opposing polar solvation term (24.57 ± 1.88 kcal/mol). These computational results suggest that PP-121 binds PVR with reasonable stability and geometric fit, supporting its use as a candidate small-molecule probe for future target validation.

**Table 2:** Binding free energies and energy components of the PVR/PP-121 complex predicted by MM/GBSA (kcal/mol).

System name	PVR / PP-121
Δ*E*_vdw_	−20.78 ± 2.37
Δ*E*_elec_	−15.16 ± 2.17
ΔG_GB_	24.57 ± 1.88
ΔG_SA_	−2.64 ± 0.12
ΔG_bind_	−14.02 ± 1.74

ΔE_vdW_: van der Waals energy; ΔE_elec_: electrostatic energy; ΔG_GB_: polar contribution to solvation; ΔG_SA_: non-polar contribution to solvation; ΔG_bind_: total binding free energy. Values are reported as mean ± standard deviation (kcal/mol), calculated from the 90–100 ns segment of the MD trajectory using MM/GBSA.

## 4 Discussion

In our study, we developed an integration pipeline that combines scRNA-seq, spatial transcriptomics, deep-learning-based histopathology and ensemble machine learning to characterize EMT-associated tissue states in gastric cancer across multiple biological scales. By analyzing approximately 250,000 single cells from two independent patient cohorts, we characterized fibroblast subpopulations with distinct spatial and survival-associated programs and localized niches in which EMT-associated, stromal and immune-related gene-set signals co-occurred. These findings supported construction of an RSF-based prognostic model based on EMT genes linked to pathology. Multiple analytical streams prioritized PVR as an EMT-associated candidate, and our loss-of-function experiments supported a tumor cell-intrinsic role for PVR in regulating gastric cancer cell proliferation and migration. However, because PVR expression in the single-cell atlas was highest in endothelial, epithelial and fibroblast compartments and low in lymphoid/plasma cells, we avoid framing PVR as a stromal immune biomarker without direct compartment-specific evidence. Docking and 100-ns molecular dynamics simulation suggested that PP-121 could serve as a candidate ligand for future biochemical testing.

One of the main themes of the study is the diversity in the fibroblast compartment in the gastric TME. Fibroblasts were separated into seven sub-lineages: Fib_APOD, Fib_COL4A1, Fib_SLPI, Fib_COL1A1, Fib_CCL4, Fib_STMN1, and Fib_S100B. Trajectory assessment through scTour placed the Fib_SLPI subpopulation toward a late-pseudotime region, suggesting an activated or specialized SLPI-high fibroblast state rather than a proven endpoint of differentiation. Because scTour and Slingshot were applied to cross-sectional scRNA-seq data, pseudotime represents an inferred ordering based on transcriptomic similarity and should not be equated with real chronological differentiation, irreversible lineage progression, or fixed cell fate. According to a previous characterization of SLPI (secretory leukocyte protease inhibitor) as a prognostic marker in gastric cancer, high expression was associated with more advanced TNM stage, presence of lymph node metastasis, and shorter five-year survival (27). It has also been characterized as a pleiotropic factor promoting the migratory and invasive behavior of colorectal cancer via AKT pathway activation, while overexpression has been associated with increased metastatic capacity in other epithelial cancers (28–30). Our finding that Fib_SLPI is spatially enriched in metastatic tissue warrants further evaluation of this activated SLPI-high fibroblast state as a prognostic biomarker and/or a microenvironmental target.

The Scissor-based analysis should be interpreted as phenotype-guided evidence for a survival-associated fibroblast program rather than as an independent demonstration that a specific fibroblast subtype causes poor prognosis. Within this framework, a COL4A1-expressing fibroblast compartment contained a substantial Scissor+ population and spatially overlapped with regions of high EMT pathway activity. COL4A1 encodes the alpha-1 chain of type IV collagen, a major basement-membrane component, and dysregulated type IV collagen remodeling has been implicated in ECM remodeling, invasion and angiogenesis in several cancers. In gastric cancer, CAF-associated matrix remodeling can create a permissive environment for tumor cell spread. These observations suggest that COL4A1-expressing fibroblast states may mark matrix-remodeling niches spatially associated with EMT activity, but they do not establish a causal effect of these fibroblasts on EMT. Cross-sectional spatial transcriptomic data measure co-localization and neighborhood structure at one tissue state and cannot determine directionality, temporal order or cell-to-cell causal regulation. The NicheNet analysis nominated candidate ligand-receptor interactions between Scissor+ fibroblasts and EMT-active cells, consistent with models in which CAFs may contribute to EMT through secreted factors such as TGF-beta, IL-6 and HGF. However, these inferred interactions require validation by higher-resolution spatial co-localization, co-culture perturbation, targeted receptor manipulation or *in vivo* functional assays. Future analyses should also test the Scissor+ signature in external cohorts and in multivariable Cox models adjusted for clinical stage and bulk stromal or immune content.

PVR/CD155 is an immunoglobulin-superfamily type I transmembrane glycoprotein. It was originally identified as the receptor mediating poliovirus cell entry, but is now recognized as a molecule with both tumor-intrinsic and immune-regulatory functions. PVR can affect cell adhesion, migration and proliferation, and it also serves as a ligand for receptors expressed on T cells and NK cells, including the activating receptor DNAM-1/CD226 and the inhibitory receptors TIGIT and CD96. The balance among these receptor interactions can shape anti-tumor immune responses, but demonstrating this mechanism in a specific tumor context requires direct evidence of compartment-specific PVR expression, spatial proximity to receptor-positive immune cells and functional perturbation.

The expression of PVR is largely low across most healthy adult tissues under physiological conditions. In contrast, high levels of PVR have been noted in several cancers, including melanoma, lung, colon, pancreas, and liver cancer, as well as gastric cancer in which higher expression often correlates with more advanced stage, aggressive phenotype and shorter survival (31). The TIGIT/PVR signaling axis is an immune checkpoint pathway, which functions in parallel to PD-1/PD-L1 signaling. In preclinical studies, combined blockade of TIGIT and PD-1 was found to have synergistic anti-tumor effect (32, 33).

Prior studies in gastric cancer and other tumors support both oncogenic and immunomodulatory roles for PVR. PVR has been reported to promote proliferation and migration in gastric cancer cells through Ras/Raf/MEK/ERK and PI3K/Akt signaling activation. Tumor-expressed PVR can also suppress NK- or T-cell cytotoxicity through TIGIT/CD96-biased signaling in some settings. In the present study, however, the strongest direct evidence comes from AGS cell knockdown and tumor-tissue staining, which support a tumor cell-intrinsic EMT-associated role. Our single-cell and spatial analyses did not establish PVR as a stromal immune biomarker: lymphoid/plasma-cell PVR detection was low, PVR correlations with TIGIT/CD226/CD96 receptor score and exhausted T-cell score were weak and section-dependent, and we did not perform spatial multiplex protein co-localization or immune-functional perturbation assays. The stromal/immune implications of PVR therefore remain hypothesis-generating and should be tested by multiplex spatial co-localization with TIGIT, CD226, CD96 and exhaustion markers, followed by compartment-specific PVR perturbation in tumor-immune or stromal-immune co-culture systems.

A major remaining limitation is that we did not perform CAF-tumor co-culture, conditioned-medium experiments, receptor perturbation, spatially resolved multiplex validation, or immune-cell functional assays to test whether Fib_COL4A1/Scissor+ fibroblasts or stromal PVR regulate PVR-expressing tumor cells or TIGIT/CD226/CD96-positive immune cells. Thus, any stromal-to-tumor or stromal-immune communication involving Fib_COL4A1, PVR, or candidate ligand-receptor pairs remains hypothesis-generating and should be tested experimentally in future studies.

Several limitations should be noted. First, the two independent single-cell datasets integrated here may underrepresent the clinical and molecular diversity of gastric cancer across disease-relevant populations and histological subtypes, including diffuse gastric cancer (DGC) and intestinal-type gastric cancer (IGC), which differ substantially in stromal and immune composition. Second, although we validated our pathology-based prognostic model in two independent cohorts, both TCGA-STAD and GSE62254 are predominantly Western patient cohorts; confirming generalizability in Asian cohorts, where gastric cancer incidence is highest, will be important. Third, Ctrl.PM did not represent healthy donor peritoneum. Although it was treated as normal-appearing peritoneal tissue from patients with peritoneal metastasis according to the source annotation, the public metadata did not provide sufficient fields to verify the exact sampling site, matched-patient status for every sample, or central pathology-negative review. Therefore, myeloid or fibroblast enrichment in Ctrl.PM should be viewed as descriptive tissue composition rather than direct evidence of a pre-metastatic niche, and future studies should include healthy or benign surgical peritoneal controls with matched histopathology. Moreover, functional experiments were performed with transient siRNA knockdown in a single cell line (AGS) in two-dimensional culture, and future work using stable knockout models, multiple cell lines, *in vivo* xenografts and patient-derived organoids will be required to validate our findings more robustly. The nomination of PP-121 was entirely based on computational modeling, and biophysical verification of binding and target specificity is still pending. Finally, the ligand-receptor interactions postulated by NicheNet require validation in experimental co-culture systems or through targeted receptor manipulation.

## 5 Conclusion

This study presents an integrative multi-omics framework — combining single-cell RNA sequencing, spatial transcriptomics, digital pathology, ensemble machine learning, and structural molecular dynamics — to characterize the EMT-associated landscape of gastric cancer. We resolved the fibroblast compartment into seven distinct sub-lineages (Fib_APOD, Fib_COL4A1, Fib_SLPI, Fib_COL1A1, Fib_CCL4, Fib_STMN1, and Fib_S100B), identifying Fib_SLPI as a metastasis-associated population and using phenotype-guided Scissor mapping to nominate a survival-associated fibroblast program enriched within COL4A1-expressing fibroblast states. Harmony integration QC supported sample-level batch mixing while retaining major cell-type marker structure, providing an additional quality-control basis for interpreting the integrated single-cell atlas. Spatial transcriptomic mapping across all available Visium sections showed that EMT pathway activity varies significantly across quantitatively defined niches and displays measurable spatial autocorrelation and EMT-niche co-localization, providing an associative spatial context for EMT-related tissue organization beyond representative images. These spatial findings support proximity and co-localization, not a causal effect of a fibroblast subtype on EMT. An optimized RSF-based prognostic model built from pathology-correlated EMT genes achieved stable, reproducible survival stratification across independent cohorts and outperformed standard clinical parameters. Cross-referencing multiple analytical streams identified PVR/CD155 as an EMT-linked candidate, and its upregulation was validated in gastric cancer cell lines and clinical tumor tissue. siRNA knockdown experiments confirmed that PVR supports proliferation, migration, and invasion in AGS cells, supporting a tumor cell-intrinsic role. Finally, structure-based docking and a 100-ns molecular dynamics simulation identified the multi-kinase inhibitor PP-121 as a candidate small-molecule PVR binder, offering a structural starting point for future targeting efforts.

## Data Availability

The original contributions presented in the study are included in the article/**Supplementary Material**. Further inquiries can be directed to the corresponding author.
