# Supplementary material for: Unraveling tumor cell heterogeneity and epithelial-mesenchymal plasticity in gastric adenocarcinoma: an integrative multi-omics framework evaluating PVR/CD155 as a tumor cell-intrinsic EMT-associated target
Source: Front Immunol. 2026 Sep 7;17:1932169. doi: 10.3389/fimmu.2026.1932169 (PMC13590775; doi:10.3389/fimmu.2026.1932169)
Supplement: Supplementary file 1 [file Table3.docx]

| **Name** | **Strand** | **Sequence (5'→3')** |
| --- | --- | --- |
| **qPCR primers for PVR** | | |
| qPVR-F | Forward | CAGTGAGCACTCAGGTACAG |
| qPVR-R | Reverse | CAGTCTCTCTCCCCTCTCAG |
| **siRNAs targeting PVR** | | |
| siRNA1 | Sense | CUGGUACCUUGGCCAGAAU(dT)(dT) |
|  | Antisense | AUUCUGGCCAAGGUACCAG(dT)(dT) |
| siRNA2 | Sense | GGAGGUGACGCAUGUGUCA(dT)(dT) |
|  | Antisense | UGACACAUGCGUCACCUCC(dT)(dT) |
| siRNA3 | Sense | GAGGAUGAAGGCAACUACA(dT)(dT) |
|  | Antisense | UGUAGUUGCCUUCAUCCUC(dT)(dT) |
