# Supplementary material for: Unraveling tumor cell heterogeneity and epithelial-mesenchymal plasticity in gastric adenocarcinoma: an integrative multi-omics framework evaluating PVR/CD155 as a tumor cell-intrinsic EMT-associated target
Source: Front Immunol. 2026 Sep 7;17:1932169. doi: 10.3389/fimmu.2026.1932169 (PMC13590775; doi:10.3389/fimmu.2026.1932169)
Supplement: Supplementary file 2 [file Table4.docx]

The supplementary data (CSV file) are available on Zenodo at https://doi.org/10.5281/zenodo.20196225
