# Supplementary material for: Unraveling tumor cell heterogeneity and epithelial-mesenchymal plasticity in gastric adenocarcinoma: an integrative multi-omics framework evaluating PVR/CD155 as a tumor cell-intrinsic EMT-associated target
Source: Front Immunol. 2026 Sep 7;17:1932169. doi: 10.3389/fimmu.2026.1932169 (PMC13590775; doi:10.3389/fimmu.2026.1932169)
Supplement: Supplementary file 3 [file Table5.docx]

Supplementary Material

# 1 Supplementary Figure and Tables

## 1.1 Supplementary Figure

Supplementary Figure 1. Single-cell quality control overview for the integrated single-cell atlas. (A) Violin plots showing the distributions of genes per cell (log10), mitochondrial transcript percentage, and UMIs per cell (log10) across sample groups: Ctrl (non-tumor gastric control), PT (primary tumor), Ctrl.PM (normal-appearing peritoneal tissue from patients with peritoneal metastasis), and PM (peritoneal metastasis). (B) UMAP embedding colored by sample/batch, illustrating sample-level mixing after Harmony integration. (C) UMAP embedding colored by tissue group (Ctrl, PT, Ctrl.PM, PM). (D) Stacked bar plot of cell-type composition (Tcs, Mye, Epi, Ecs, Plasma, Bcs, Fib, Mast) across individual samples, grouped by tissue type. (E) Box plots comparing cell-type kNN purity and sample mixing (iLISI) between PCA (pre-integration) and Harmony (post-integration) embeddings, demonstrating improved batch mixing while preserving cell-type marker identity.

Supplementary Figure 2 | Spatial Visium quality control, SpaTopic diagnostics, and EMT activity. (A) Quality-control summaries for four Visium sections (GC1, GC4, GC7, and GC9). Bars show the number of spots retained for analysis and the section-level median numbers of detected genes and unique molecular identifiers (UMIs) per spot, together with the median mitochondrial transcript fraction. The analyzed spot numbers were 3,834, 4,244, 1,882, and 3,491, respectively. (B) Section-level diagnostics for the retained 10-topic SpaTopic model, including the fraction of spots for which the assigned CellTopic had the maximum loading, the median loading of the assigned dominant topic, and the median normalized topic entropy. (C) Distributions of spot-level epithelial-mesenchymal transition (EMT) scores across SpaTopic-defined spatial niches. EMT scores were calculated as the mean standardized expression of detected MSigDB Hallmark EMT genes within each section. Violin plots show score distributions; boxes indicate the interquartile range, white points indicate medians, and whiskers extend to 1.5 times the interquartile range. EMT activity differed significantly among niches in all sections (Kruskal–Wallis test: GC1, H = 1556.68, q < 1 × 10-300; GC4, H = 2009.44, q < 1 × 10-300; GC7, H = 421.16, q = 7.45 × 10-90; GC9, H = 2068.66, q < 1× 10-300). (D) Spatial distributions of spot-level EMT activity. Color scales were determined separately for each section using the 1st–99th percentile range. Scale bars, 0.5 mm. (E) Spearman spatial cross-correlations between the EMT score of each spot and the proportion of neighboring spots assigned to each niche within a 200-μm radius. Values denote Spearman’s ρ; blank cells indicate niches not identified in the corresponding section. (F) Spatial autocorrelation of EMT activity quantified using Moran’s I and Geary’s C with a 200-μm neighborhood radius. Significance was assessed using 99 permutations, and P values were adjusted using the Benjamini–Hochberg method. All four sections exhibited significant positive spatial clustering of EMT activity.

Supplementary Figure 3. SpaTopic-derived spatial niche composition across Visium sections GC1, GC4, GC7, and GC9. (A) Stacked bar plots showing the proportion of spots assigned to each spatial niche category (Epithelial, Fibroblast, Endothelial, Immune, Infiltration, Myeloid, B cell, Plasma, Mast) per section. (B) Heatmap of CellTopic loading scores disaggregated by niche category, illustrating the dominant CellTopic contributions within each niche.

Supplementary Figure 4. Single-cell distribution and spatial immune-checkpoint correlations of PVR/CD155. (A) Specimen-level heatmap showing the percentage of fibroblasts classified as Scissor+ overall and within seven fibroblast subclusters. Scissor+ denotes fibroblast states positively associated with mortality risk in the Scissor Cox model. Columns represent profiled specimens, with the upper annotation indicating Ctrl, Ctrl.PM, PM, and PT groups; the color scale is capped at ≥30%. (B) Dot plot showing PVR expression across major cell types in the integrated single-cell atlas. Dot size represents the percentage of cells with detectable PVR expression, and color represents average expression. Ecs, endothelial cells; Epi, epithelial cells; Fib, fibroblasts; Mye, myeloid cells; Tcs, T cells; Bcs, B cells. (C) Percentage of PVR-positive cells in each cell type. PVR was detected most frequently in endothelial cells (13.8%), epithelial cells (12.9%), and fibroblasts (9.9%), with lower detection frequencies in myeloid cells (3.5%), mast cells (0.8%), T cells (0.3%), plasma cells (0.3%), and B cells (0.2%). (D) Spot-level Spearman correlations between PVR expression and individual immune-checkpoint or T-cell exhaustion markers, the TIGIT/CD226/CD96 receptor score, the exhausted T-cell score, and the T-cell score across four Visium sections. Colored points represent individual sections, and horizontal lines indicate medians across sections. The receptor score was calculated as the mean expression of TIGIT, CD226, and CD96; the exhausted T-cell score comprised PDCD1, CTLA4, HAVCR2, LAG3, TOX, ENTPD1, CXCL13, and TIGIT; and the T-cell score comprised CD3D, CD3E, CD8A, and CD8B. (E) Spearman correlations between spot-level PVR expression and the mean receptor or exhausted T-cell score of neighboring spots within a 200-μm radius. Colored points denote individual sections and horizontal lines indicate cross-section medians. (F) Spatial distributions of PVR expression in GC1, GC4, GC7, and GC9. (G) Spatial distributions of the TIGIT/CD226/CD96 receptor score. (H) Spatial distributions of the PVR-neighbor exhausted T-cell colocalization score, calculated as the product of within-section standardized PVR expression and the standardized mean exhausted T-cell score of neighboring spots within 200 μm. Spatial color scales were determined separately for each section using the 1st–99th percentile range. Scale bars, 0.5 mm. These analyses identify weak and section-dependent spatial associations and do not establish compartment-specific or causal immune regulation by PVR.

Supplementary Figure 5. Molecular docking of candidate compounds against PVR/CD155. Predicted binding modes of seven candidate compounds docked to the AlphaFold-derived extracellular structure of human PVR/CD155 using AutoDock Vina. The compounds and predicted docking scores were: (A) (2E)-N-(4-((3-bromophenyl)amino)quinazolin-6-yl)-4-(dimethylamino)but-2-enamide, −5.893 kcal/mol; (B) 1-(1-(3-aminophenyl)-3-tert-butyl-1H-pyrazol-5-yl)-3-phenylurea, −6.222 kcal/mol; (C) 1-(1-methylethyl)-3-(quinolin-6-yl)-1H-pyrazolo[3,4-d]pyrimidin-4-amine, −6.421 kcal/mol; (D) 1-cyclobutyl-3-(3,4-dimethoxyphenyl)-1H-pyrazolo[3,4-d]pyrimidin-4-amine, −6.012 kcal/mol; (E) 4-((4-methyl-1-piperazinyl)methyl)-N-(3-((4-(3-pyridinyl)-2-pyrimidinyl)amino)phenyl)benzamide, −8.219 kcal/mol; (F) KX-01, −7.637 kcal/mol; and (G) N-(4-phenylaminoquinazolin-6-yl)acrylamide, −5.493 kcal/mol. PVR is displayed as a light-blue cartoon, ligands and interacting residues as sticks, and dashed lines or colored annotations indicate predicted hydrogen-bond, hydrophobic, and π-mediated interactions. More negative docking scores indicate more favorable predicted binding. PP-121, which had a docking score of −6.585 kcal/mol and was selected for subsequent molecular-dynamics analysis, is presented in the main figure.

Supplementary Figure 6. PVR/CD155 knockdown alters EMT-marker expression in AGS gastric cancer cells. RT-qPCR analysis of PVR/CD155 and epithelial-mesenchymal transition markers in untreated AGS cells (Control), cells transfected with a non-targeting siRNA (NC), and cells transfected with three independent PVR/CD155-targeting siRNAs (siCD155-1, siCD155-2, and siCD155-3). Relative mRNA expression was calculated using the 2−ΔΔCt method and normalized to the untreated control. (A) PVR/CD155 expression, confirming knockdown efficiency. (B) E-cadherin/CDH1 expression. (C) N-cadherin/CDH2 expression. (D) Vimentin/VIM expression. (E) SNAIL/SNAI1 expression. PVR/CD155 silencing increased the epithelial marker E-cadherin and reduced the mesenchymal markers N-cadherin, vimentin, and SNAIL. Data are presented as mean ± SD of triplicate measurements. Statistical comparisons were performed against the NC group; ***P < 0.001.

Supplementary Figure 7. Knockdown of the target gene by siRNA2 and siRNA3 suppresses cell migration. (A) For the Transwell migration assay, the number of migrated cells per field should be quantified and presented as a bar graph with standard deviation and statistical significance markers (mean ± SD; *p* < 0.05, **p* < 0.01, ***p* < 0.001; n = 3 per group). (B) For the wound‑healing assay, the percentage of wound closure should be calculated at each time point and plotted as a bar graph (mean ± SD; *p* < 0.05, **p* < 0.01, ***p* < 0.001; n = 3 per group).

## 1.2 Supplementary Tables

Supplementary Table 1. ScRNA-seq QC sample counts.

Supplementary Table 2. Single-cell QC and Harmony integration metrics.

Supplementary Table 3. Fibroblast subcluster marker genes.

Supplementary Table 4. Spatial revision QC statistics.

Supplementary Table 5. MSigDB Hallmark EMT gene set.

Supplementary Table 6. ML hyperparameters.

Supplementary Table 7. Scissor NicheNet definitions.

Supplementary Table 8. Primer and siRNA sequences used in this study.

Supplementary Table 9. ResNet50-derived deep learning features from TCGA-STAD whole slide images. The supplementary data (CSV data file) are available at <https://doi.org/10.5281/zenodo.20196225>.

Supplementary Table 10. CellProfiler morphological features from TCGA-STAD WSI.
